# Molecular phenotyping of a UK population: defining the human serum metabolome

**DOI:** 10.1007/s11306-014-0707-1

**Published:** 2014-07-25

**Authors:** Warwick B. Dunn, Wanchang Lin, David Broadhurst, Paul Begley, Marie Brown, Eva Zelena, Andrew A. Vaughan, Antony Halsall, Nadine Harding, Joshua D. Knowles, Sue Francis-McIntyre, Andy Tseng, David I. Ellis, Steve O’Hagan, Gill Aarons, Boben Benjamin, Stephen Chew-Graham, Carly Moseley, Paula Potter, Catherine L. Winder, Catherine Potts, Paula Thornton, Catriona McWhirter, Mohammed Zubair, Martin Pan, Alistair Burns, J. Kennedy Cruickshank, Gordon C. Jayson, Nitin Purandare, Frederick C. W. Wu, Joe D. Finn, John N. Haselden, Andrew W. Nicholls, Ian D. Wilson, Royston Goodacre, Douglas B. Kell

**Affiliations:** 1 Faculty of Engineering and Physical Sciences, School of Chemistry, Manchester Institute of Biotechnology, The University of Manchester, Manchester, M1 7DN UK; 2 Faculty of Engineering & Physical Sciences, Manchester Centre for Integrative Systems Biology, Manchester Institute of Biotechnology, The University of Manchester, Manchester, M1 7DN UK; 3 Faculty of Medical and Human Sciences, Centre for Endocrinology and Diabetes, Institute of Human Development, The University of Manchester, Manchester, UK; 4 Centre for Advanced Discovery and Experimental Therapeutics (CADET), Central Manchester University Hospitals NHS Foundation Trust, Manchester Academic Health Sciences Centre, Manchester, M13 9WL UK; 5 School of Biosciences, University of Birmingham, Edgbaston, Birmingham B15 2TT, UK; 6 Department of Medicine, Katz Group Centre for Pharmacy & Health, University of Alberta, Edmonton, AB T6G 2R3 Canada; 7 Faculty of Engineering & Physical Sciences, School of Computer Science, The University of Manchester, Manchester, M13 9PL UK; 8 Faculty of Medical & Human Sciences, Institute of Inflammation and Repair, Salford Royal Dermatopharmacology, The University of Manchester, Manchester, M6 8HD UK; 9 Clinical & Cognitive Neurosciences, Faculty of Medical & Human Sciences, Institute of Brain, Behaviour & Mental Health, The University of Manchester, Manchester, M13 9PT UK; 10 Faculty of Medical and Human Sciences, Institute of Cancer Sciences, Christie Hospital, The University of Manchester, Manchester, M20 4BX UK; 11 Faculty of Medical & Human Sciences, Institute of Human Development, The University of Manchester, Manchester, M13 9WL UK; 12 Neurology and Gastrointestinal Centre of Excellence for Drug Discovery, GlaxoSmithKline, Third Avenue, Harlow, Essex, CM19 5AW UK; 13 Faculty of Medical & Human Sciences, Institute of Cardiovascular Sciences, The University of Manchester, Manchester, M13 9PT UK; 14 St Thomas’ Hospital, King’s College, University of London & King’s Health Partners, London, SE1 9NH UK; 15 Investigative Preclinical Toxicology, GlaxoSmithKline, David Jack Centre for Research and Development, Park Road, Ware, Hertfordshire, SG12 0DP UK; 16 Faculty of Medicine, Department of Surgery & Cancer, Sir Alexander Fleming Building, Imperial College London, London, SW7 2AZ, UK; 17 DMPK Innovative Medicines, AstraZeneca, Cheshire, SK10 4TF UK

**Keywords:** Human serum, Metabolic phenotyping, UK population, Mass spectrometry, Clinical biochemistry

## Abstract

Phenotyping of 1,200 ‘healthy’ adults from the UK has been performed through the investigation of diverse classes of hydrophilic and lipophilic metabolites present in serum by applying a series of chromatography–mass spectrometry platforms. These data were made robust to instrumental drift by numerical correction; this was prerequisite to allow detection of subtle metabolic differences. The variation in observed metabolite relative concentrations between the 1,200 subjects ranged from less than 5 % to more than 200 %. Variations in metabolites could be related to differences in gender, age, BMI, blood pressure, and smoking. Investigations suggest that a sample size of 600 subjects is both necessary and sufficient for robust analysis of these data. Overall, this is a large scale and non-targeted chromatographic MS-based metabolomics study, using samples from over 1,000 individuals, to provide a comprehensive measurement of their serum metabolomes. This work provides an important baseline or reference dataset for understanding the ‘normal’ relative concentrations and variation in the human serum metabolome. These may be related to our increasing knowledge of the human metabolic network map. Information on the Husermet study is available at http://www.husermet.org/. Importantly, all of the data are made freely available at MetaboLights (http://www.ebi.ac.uk/metabolights/).

**Electronic supplementary material:**

The online version of this article (doi:10.1007/s11306-014-0707-1) contains supplementary material, which is available to authorized users.

## Introduction

The biochemical composition of human cells, tissues and biofluids is highly complex, and their integrative and dynamic interactions (termed the interactome) defines function and phenotype (Vidal et al. 2011). Of these biochemicals, small molecule metabolites are involved in many important processes, from acting as the building blocks for larger biochemicals and structures, in regulation of biochemical processes, and within metabolism to generate essential cellular components (Dunn et al. 2011). The quantitative collection of metabolites in a biological system is defined as the metabolome (Oliver et al. 1998), with sample-specific metabolomes differing in composition both qualitatively and quantitatively. For fundamental reasons, the metabolome is expected [e.g. Kell (2004, 2006a, b), Kell and Westerhoff (1986)] and is indeed found (Raamsdonk et al. 2001), to amplify changes observed in the transcriptome and proteome. The holistic study of the quantitative complement of metabolites in humans provides a sensitive and dynamic snapshot of the human metabolic phenotype (Dunn et al. 2011) [also referred to as the metabotype (Gavaghan et al. 2000)]. Knowledge of variations in metabotype may be applied in disease risk prediction and diagnosis, in understanding molecular pathophysiology, in interpreting the influence of our environment and lifestyle and in the development and assessment of drug efficacy, toxicity and adverse drug reactions. Metabolomics thus has an important role to play in personalized and stratified medicine (Nicholson et al. 2012; van der Greef et al. 2006).

Both genetics and the environment contribute significantly to human function and phenotype. Recent studies have sought to relate the influence of the genetic fingerprint on metabolism, including through the application of genome-wide association (GWAS)-metabolomics studies (Suhre and Gieger 2012; Suhre et al. 2011). These and other studies have shown the importance of applying metabolomics, alone or as part of integrated multi-omic studies to investigate human phenotypes. The use of ^1^H NMR spectroscopy to analyse urine samples, collected in large scale epidemiological studies, has revealed interesting trends between populations and provided new biomarkers, related for example to blood pressure differences between individuals and populations (Holmes et al. 2008; Yap et al. 2010). However, whilst robust and precise, ^1^H NMR spectroscopy does not access the whole metabolome and the use of other metabolite profiling technologies such as gas chromatography–mass spectrometry (GC–MS) and ultra performance liquid chromatography–mass spectrometry (UPLC–MS) offer excellent opportunities for expanding metabolome coverage due to the prior chromatographic separation of the many thousands of small molecules estimated via analysis of the human metabolic network (Kell and Goodacre 2014; Thiele et al. 2013) to be in the human metabolome, followed by sensitive MS-based detection. A small scale study to characterize the human serum metabolome has been performed in <150 subjects (including quantification of a subset of metabolites). This study, which employed multiple analytical platforms highlighted the importance of this strategy to broaden the coverage of the metabolome and provided the first experimentally-derived serum metabolome database (Psychogios et al. 2011). However, it is only recently that technological and methodological advances that compensate for unavoidable instrumental drift (Begley et al. 2009; Dunn et al. 2011; Zelena et al. 2009) and provide high quality data have allowed us to study the large populations and numbers of metabolites needed (Broadhurst and Kell 2006) in epidemiological studies with these non-targeted MS-based techniques. Studies applying targeted assays to study low hundreds of metabolites have also been reported (Cheng et al. 2012; Yu et al. 2012).

Here we present data from The Husermet project (http://www.husermet.org/) which has applied non-targeted chromatography-mass spectrometry platforms to study the hydrophilic and lipophilic metabolic complement of serum samples obtained in a large (*n* = 1,200) investigation of the phenotype of a ‘healthy’ UK adult population. This required the development of substantive methods able to deal with long-term drift observed in such instrumentation. Serum samples were collected from normal healthy adults (that is to say, with no known disease at the time of sampling) of between 19 and 81 years of age over a 4-year period. We describe the variations and the influence of age, gender, BMI, blood pressure and smoking on the human serum metabolome, and the correlation of clinical chemistry measures with hydrophilic and lipophilic metabolites.

## Results and discussion

### Metabolic phenotyping of 1,200 subjects from a UK population

More than 3,000 serum samples were collected at three separate UK locations across a 4-year period, applying the same standard operating procedure (SOP) at all sites. 1,200 serum samples were selected for a first-pass of data acquisition. GC–MS and UPLC–MS in positive (UPLC–MS(+)) and negative ion (UPLC–MS(−)) ion modes were applied as complementary analytical platforms to profile a diverse range of hydrophilic and lipophilic metabolites present in the serum of 1,200 adult subjects from the UK in the age range of 19–81 years; at the time of sampling all subjects were defined as ‘healthy’, with no diagnosis of any disease. Data were acquired across 11 months in 10 different analytical experimental batches; each batch was composed of a single serum sample from 120 subjects and analysed across a five-day period. Each batch included the periodic analysis of a pooled quality control (QC) sample) to allow analytical variation to be measured quantitatively within and between these analytical experiments (Dunn et al. 2012). The same pooled QC sample was applied for all analytical experimental runs.

Following data pre-processing to construct a robust dataset, 126, 2178 and 2280 *metabolite features* were detected by GC–MS, UPLC–MS(+) and UPLC–MS(−), respectively; due to multiple adducts/fragments etc. during electrospray ionisation (Brown et al. 2009) more than 1,500 metabolites are estimated as being detected. All of these metabolites were detected reproducibly across all analytical experimental batches in a periodically analysed (every 5th injection) single pooled QC sample; this quantifies the variation introduced by sample preparation, data acquisition and data pre-processing. The criterion applied to define reproducible detection was relative standard deviation (RSD) less than 20 % for UPLC–MS and RSD less than 30 % for GC–MS, calculated after signal correction [see Dunn et al. (2011)]. Classes of metabolites detected included amino acids (GC–MS), organic acids (GC–MS), carbohydrates (GC–MS), fatty acids (GC–MS and UPLC–MS), peptides (UPLC–MS), acyl glycerides (UPLC–MS), sphingolipids (UPLC–MS), steroids including vitamin D metabolites (UPLC–MS) and glycerophospholipids (UPLC–MS), representing a diverse set of metabolic pathways and regulatory processes. This allowed many different areas of metabolism and biological function to be investigated simultaneously, so as to identify their importance with regard to the human ‘healthy’ population phenotype. This approach is in contrast to targeted studies that focus on small segments of metabolism or just a few metabolite classes. Additionally, a variety of exogenous metabolites were also detected including drugs and their metabolic products (e.g. paracetamol, (acetaminophen)). By applying linear discriminant analysis, we concluded that no metabolic differences were observed that could be related to time differences in acquiring the analytical data (see Supplementary Fig. 1), showing for the first time that a metabolome-wide study of large sample sets derived from the human population could be profiled reproducibly via chromatography-mass spectrometry platforms over a period of 11 months. A range of standard clinical chemistry measurements was also performed for all 1,200 subjects (23 assays in total including lipids (LDLC, CHOL, HDLC, TRIG), enzyme concentrations (PLT, ALK, AST, ALT, GGT, ALP, LDH), metabolites (glucose, creatinine, urea), ions (Ca, K, Na, phosphate), blood components (WBC, RBC, HAEM, TBIL) and total protein and albumin) and provided the ability to relate changes in these assays applied in routine clinical use to metabolic pathways and associated mechanisms. All metabolite data and associated demographic/clinical metadata are available at the publically available metabolomics data repository MetaboLights (http://www.ebi.ac.uk/metabolights/; study identifier MTBLS97). The clinical characteristics of the cohort discussed here are provided in Table 1.

**Table 1:** Clinical characteristics of the cohort studied defining median and inter-quartile range

Characteristic	
Gender (male:female)	701:490^a^
Age (median, IQR)	48.0 (40.0,60.0)^b^
BMI (median, IQR)	25.63 (23.20,28.71)^b^
Smokers (non:ex:current)	502:163:176^c^
SBP (median, IQR), mmHg	125 (115,137)^d^
DBP (median, IQR), mmHg	76 (70,83)^d^
GLUC (median, IQR), mmol L^−1^	4.71 (4.20,5.30)^e^
CHOL (median, IQR), mmol L^−1^	5.10 (4.30,5.80)^f^
TRIG (median, IQR), mmol L^−1^	1.18 (0.80,1.80)^g^
HDLC (median, IQR), mmol L^−1^	1.26 (1.00,1.50)^h^
LDLC (median, IQR), mmol L^−1^	3.2 (2.54,3.77)^i^

^a^ Data not available for 8 subjects

^b^ Data not available for 4 subjects

^c^ Data not available for 355 subjects

^d^ Data not available for 179 subjects

^e^ Data not available for 175 subjects

^f^ Data not available for 262 subjects

^g^ Data not available for 326 subjects

^h^ Data not available for 347 subjects

^i^ Data not available for 360 subjects

### Variability in relative metabolite concentrations

**Fig. 1:**
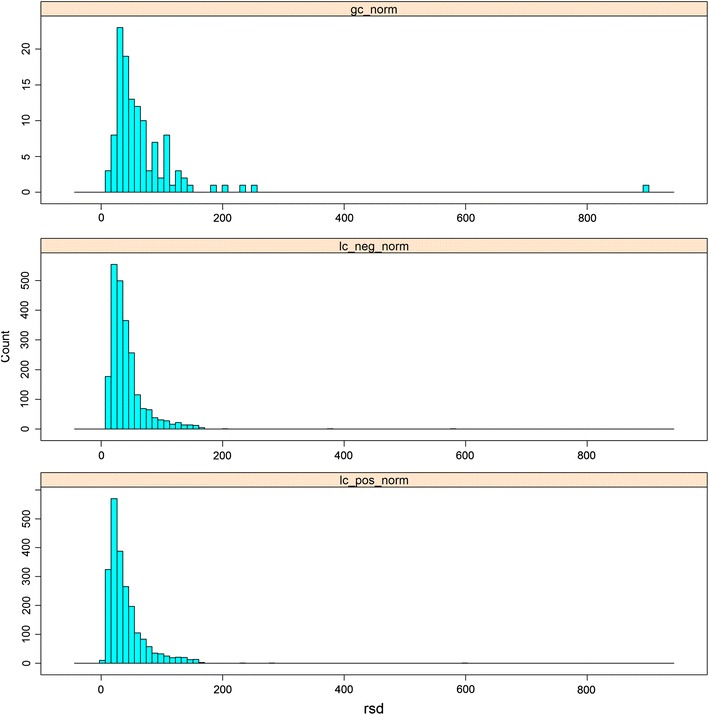
The distribution of relative standard deviations defining the inter-subject variability in metabolite relative concentrations for each analytical platform applied, following signal correction. The data are shown as distribution plots. *Top plot* GC–MS, *middle plot* UPLC–MS(−), *bottom plot* UPLC–MS(+)

The relative concentrations of metabolites were investigated to derive the cumulative variation associated with background/baseline genetic and environmental influences. The distribution of variation associated with inter-subject variability [as calculated as the relative standard deviation (RSD)] for all 1,200 subjects following signal correction) is shown in Fig. 1. The distribution is skewed to lower RSD values; one interpretation of this is that the serum metabolome is comparatively tightly regulated in “healthy” populations (i.e., subjects with no diagnosed disease at the time of sampling). This could reasonably be expected, with a greater variation observed in the human urine metabolome (Bouatra et al. 2013), a biofluid composed of metabolites that are being excreted from the body. Of course, if the inter-subject variability is equivalent to the technical variability measured by replicate analysis of the same quality control (QC) sample then the metabolite feature contains no biological information. For GC–MS, UPLC–MS(+) and UPLC–MS(−), respectively, 7 of 126, 71 of 2,181 and 42 of 2,283 *metabolic features* were observed to have an inter-subject RSD/QC RSD <1.5; thus the overwhelming majority of metabolite features reported contain biological information and those metabolite features with a value less than 1.5 were removed from further analyses.

In this distribution (see Fig. 1), those metabolites showing a high variability between subjects describe inter-subject variability which is likely to be caused by environmental and genetic variation. Caffeine showed an RSD greater than 200 % and salicylic acid, probably derived as a result of aspirin use but possibly also via tobacco, had an RSD >800 %, whilst N-methylpyrrolidinine (used in the formulation of drug vehicles) had an RSD of 550 %; such analytes thus show variation related to consumption of pharmaceutical drugs and/or specific food components. Trehalose varied by greater than 200 %, suggesting significant variation in glucose usage and storage properties, albeit trehalose is also used as a food additive. Tetradecanoic, hexadecenoic and eicosanoic acids all had variances greater than 100 %. Oxidised longer chain fatty acids and acyl carnitines also showed higher variations—potentially a sign of oxidative stress or changes in energy production in the body. Glycerophosphoethanolamines and a small peptide (*γ*-*glutamyl*-*l*-*isoleucine or γ*-*glutamyl*-*l*-*leucine*) also show high variation. By contrast, the aromatic amino acids (tryptophan, phenylalanine and tyrosine) all showed a low degree of inter-subject variability.

### Metabolite-metabolite correlations

**Fig. 2:**
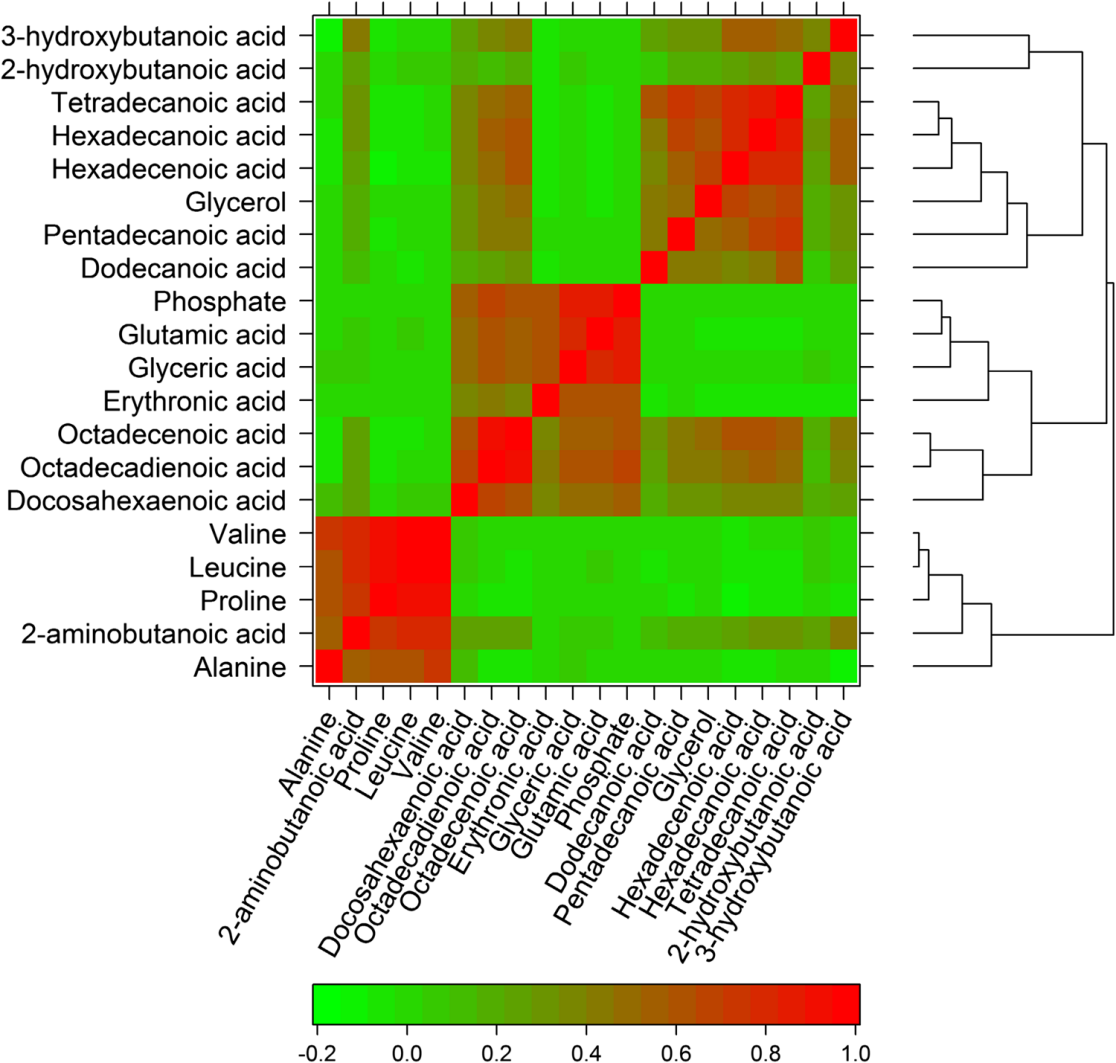
Heatmap with dendrogram of correlation network for metabolites detected by GC–MS. The twenty unique metabolites with one or more of the highest correlations are depicted. The *lower bar* represents the *colour code* of coefficients from pairwise Pearson’s correlations (Color figure online)

Metabolites do not operate in isolation but through a complex network of interactions, with metabolism being one network, though other networks are observed in biological systems (Camacho et al. 2005), especially through correlation of non-neighbour metabolites indicating their involvement in regulatory pathways [see e.g. Kotze et al. (2013)]. We note also that as reported in Camacho et al. (2005) without clear metabolite linkage, correlations should be treated with caution as correlation does not necessarily equate to causation. To highlight these complex networks we illustrate the 20 metabolites for GC–MS that show the highest pairwise Pearson’s correlations. Where a metabolite was detected as more than one ‘metabolic feature’, only one ‘feature’ has been included in Fig. 2, the feature with the higher correlation coefficient. The data show the expected correlations between leucine and valine (both involved in branched chain amino acid metabolism) and between different fatty acids and glycerol (related to glycerolipid and glycerophospholipid metabolism). However, and unexpectedly, proline was also correlated with leucine and valine, and phosphate with fatty acids. Assessing the UPLC–MS data (Supplementary Fig. 2) we detected expected correlations between fatty acids and oxidized fatty acids, between different sphingolipids, between fatty acids and sphingolipids, between different lyso-glycerophospholipids, between different diacylglycerides and between diacylglycerides and sphingolipids.

### The effect of sample size

**Fig. 3:**
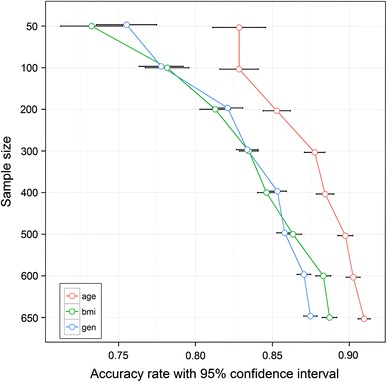
Classification analysis to assess sample size effects. The accuracy rate of discrimination with 95 % confidence intervals for data acquired applying UPLC–MS(+) for the three parameters of age (age <50 vs. age >65), BMI (BMI <25 vs. BMI >30) and gender (male vs. female). A Random Forest (RF) classifier was employed and 100 bootstrap sample sets were used for the assessment of classification accuracy

It is becoming increasingly evident that many biological studies are underpowered with regard to their ability to come to a robust and statistically significant and justifiable biological conclusion (Broadhurst and Kell 2006; Button et al. 2013; Dunn et al. 2011; Dunn et al. 2012; Ioannidis 2005; Ioannidis and Panagiotou 2011). It is obvious that sample size in metabolomic studies is an important aspect of experimental design, especially in terms of applying metabolites as predictive biomarkers. Although these issues have been addressed in theory [see Xia et al. (2013) for a detailed discussion], to our knowledge, no previous large-scale studies have assessed the influence of sample size. Thus, we studied the effect of sample size in terms of the prediction power of classification and the consistency of feature selection. The experimental design was to divide the whole sample population into several subsets for classification and feature selection. The results of these subsets are used to select the smallest subset which has an acceptable performance, comparing this with the whole sample population in both classification and feature selection. Three groups, viz. age, gender and BMI for the three analytical platforms, have been used to evaluate the effects of sample size. Sample size is defined as the sum of samples in both classes in a binary classification and in this study the number of samples in each class was not equivalent (see Supplementary Table 1). In an ideal study the number of samples in each class would be balanced. Figure 3 shows the prediction accuracy using Random Forests (RF) with a 95 % confidence interval in the three groups (age, gender and BMI) for UPLC–MS positive ion mode. At low sample sizes the prediction accuracy was variable, but as the sample size was increased the median accuracy also increased with concomitant decrease in variation. These data showed that a sample size of 600 was appropriate to achieve similar results to those of the whole sample population with *the current dataset* where we are looking for general (i.e. not disease-specific) changes and where the variation is expected to be lower than that for the comparison of two populations such as ones that are ‘healthy’ and ‘diseased’. A previous study based on NMR data has shown that sample sizes of low thousands of subjects offer sufficient statistical precision to detect biomarkers quantifying predisposition to disease, a different assessment to the one we have performed above (Nicholson et al. 2011). We emphasise that this highlights the requirement to include hundreds of samples in these types of studies but does not suggest that a sample size of 600 is appropriate for all studies [for detailed discussions on this subject see Xia et al. (2013)]. However, the trends observed for all analytical platforms suggested a higher sample size would still slightly increase the prediction accuracy. The same trends were also seen with UPLC–MS(−) as well as for GC–MS. Classification results with RF and Support Vector Machine (SVM) classifiers for all three platforms and the effects of sample size on feature selection are shown in Supplementary Figs. 3 and 4.

### Metabolic characteristics of this UK population

Metabolic characteristics of this subset of the UK population are discussed below. Results of data analysis performed applying consensus feature selection as described in the methods section and associated with the discussions related to gender, age, BMI, blood pressure and smoking are available. The results of two-way analysis of variance (ANOVA) and their post hoc analysis by Tukey’s HSD (“honestly significant difference”) test are available and are summarized, where appropriate, below. All data analysis results are available in supplementary material files 2–4. Where no results for two-way ANOVA are included, metabolites have been defined as biologically important by applying consensus feature selection protocol, but two-way ANOVA has shown no statistical significance with a ‘critical’ *p* < 0.05 [cf. Broadhurst and Kell (2006)]. Similarly interactions between main effects are only discussed if significant.

### Gender

**Fig. 4:**
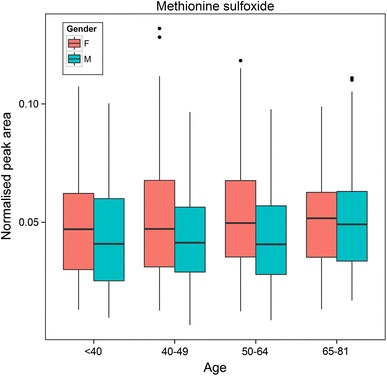
A boxplot showing the distribution of methionine sulfoxide for males and females across different age categories. For *each box*, the *central line* is the median, the edges of the *box* are the upper and lower quartiles, the whiskers extend the *box* by a further ±1.5 × interquartile range (IQR), and outliers (>1.5 × IQR) are plotted as individual points. Data were analysed using 2-way ANOVA showing a significant difference between males and females, [*F*(1,901) = 20.3, *p* = 7.7 × 10^−6^]. There was no significant difference between age categories and no significant interaction between gender and age categories

Two-way ANOVA was performed using Gender (male, female) and Age (four grouped categories: <40, 40–49, 50–64 and >64 years) as the main effects. Many differences in the serum metabolome were observed when comparing the metabolic profiles of males and females. A number of these had been observed previously highlighting the robustness of our study; these included 4-hydroxyphenyllactic acid [*F*(1,1123) = 245.1, *p* = 3.9 × 10^−50^], creatinine, citrate, urate [*F*(1,1092) = 512.3, *p* = 2.6 × 10^−93^], glycerol [*F*(1,1081) = 93.7, *p* = 2.6 × 10^−21^], hexadecenoic acid [*F*(1,1097) = 62.8, *p* = 5.5 × 10^−15^] and tyrosine (Kochhar et al. 2006; Lawton et al. 2008; Slupsky et al. 2007). For glycerol, there was also a significant difference between age categories [*F*(3,1081) = 3.1, *p* = 1.1 × 10^−12^]. Tukey post hoc test showed that, independent of gender, comparisons of age categories <40 vs. 40–49 (*p* = 0.0005), <40 vs. 50–64 (*p* = 9.1 × 10^−12^), <40 vs. 65–81 (*p* = 1.8 × 10^−8^), 40–49 vs. 50–64 (*p* = 0.004) and 40–49 vs. 65–81 (*p* = 0.03) were significant using a critical *p* value of 0.05. There was also a significant interaction between gender and age categories for urate [*F*(3,1092) = 4.8, *p* = 0.002], glycerol [*F*(3,1081) = 2.8, *p* = 0.039] and hexadecenoic acid [*F*(3,1097) = 4.7, *p* = 0.003]. In our study, 4-hydroxyphenyllactic acid was found to be higher and tyrosine lower in males. Both of these metabolites are structurally related and these differences may reflect differences in gut microfloral co-metabolism, or the effects of alcohol consumption (Liebich and Pickert 1985). However, we observed a multitude of other robust changes related to gender also. Eight diacylglycerides were observed to be higher in relative concentration in the serum of women compared to men including DG(44:6) [*F*(1,808) = 276.5, *p* = 1.3 × 10^−53^] and DG(46:2) [*F*(1,848) = 206.1, *p* = 5.3 × 10^−42^]). For DG(46:2) there was also a significant difference between age categories [*F*(3,848) = 5.8, *p* = 0.0006] and a significant interaction between gender and age categories [*F*(3,848) = 7.5, *p* = 6.0 × 10^−5^]. Tukey post hoc test showed that, independent of gender, comparisons of age categories <40 vs. 65–81 (*p* = 0.002) and 50–64 vs. 65–81 (*p* = 0.0009) were significant using a critical *p*-value of 0.05. Four fatty acids (for example, hexadecenoic acid as shown above) and thirteen glycerophospholipids (for example, PC(36:2) [*F*(1,1103) = 224.8, *p* = 2.2 × 10^−46^]) showed the same trend as diacylglycerides. PC(36:2) also showed a significant difference between age categories [*F*(3,1103) = 3.4, *p* = 0.02] and a significant interaction between gender and age categories [*F*(3,1103) = 4.5, *p* = 0.004]. Tukey post hoc test showed that, independent of gender, comparisons of age categories <40 vs. 40–49 (*p* = 0.02) was significant using a critical *p*-value of 0.05. Serum creatinine relative concentrations were observed to be higher in females than males and, when integrated with higher phosphate levels, might suggest greater breakdown of creatine phosphate in muscles in females. Caffeine relative concentrations were higher in women [*F*(1,847) = 38.3, *p* = 9.6 × 10^−10^] perhaps reflecting coffee/tea/chocolate consumption, as was 2-aminomalonic acid [*F*(1,1048) = 87.6, *p* = 4.8 × 10^−20^] which has been associated with atherosclerotic plaques (Rupérez et al. 2012) and renal failure (Mao et al. 2008). For caffeine [*F*(3,847) = 9.3, *p* = 5.0 × 10^−6^] and 2-aminomalonic acid [*F*(3,1048) = 3.6, *p* = 0.01] there was also a significant difference between age categories and a significant interaction between gender and age categories for caffeine [*F*(3,847) = 6.3, *p* = 0.0003] and 2-aminomalonic acid [*F*(3,1048) = 24.3, *p* = 3.5 × 10^−15^]. Tukey post hoc test for caffeine showed that, independent of gender, comparisons of age categories <40 vs. 40–49 (*p* = 8.2 × 10^−5^), <40 vs. 50–64 (*p* = 0.0002) and <40 vs. 65–81 (*p* = 1.4 × 10^−5^) were significant using a critical *p*-value of 0.05. Tukey post hoc test for 2-aminomalonic acid showed that, independent of gender, comparisons of age categories <40 vs. 50–64 (*p* = 0.03) and 40–49 vs. 50–64 (*p* = 0.03) were significant using a critical *p*-value of 0.05. Three glycerol-like metabolites (glyceric acid [*F*(1,1107) = 9.1, *p* = 0.003], glycerol [*F*(1,1081) = 93.7, *p* = 2.6 × 10^−21^] and glycerol-3-phosphate [*F*(1,1127) = 11.8, *p* = 0.0006]) were present in greater amounts in the serum of women compared to men, suggesting differences in glycerol metabolism and potentially related to differences in the rate of glycerolipid and glycerophospholipid synthesis. For glycerol [*F*(3,1081) = 20.1, *p* = 1.1 × 10^−12^] and glyceric acid [*F*(3,1107) = 6.8, *p* = 0.0001] there was also a significant difference between age categories. There was also a significant interaction between gender and age categories for glycerol [*F*(3,1081) = 2.8, *p* = 0.04] and glycerol-3-phosphate [*F*(3,1127) = 8.7, *p* = 1.1 × 10^−5^]. Tukey post hoc tests showed that, independent of gender, comparisons of age categories for glycerol [<40 vs. 40–49 (*p* = 0.0005), <40 vs. 50–64 (*p* = 9.1 × 10^−12^), <40 vs. 65–81 (*p* = 1.8 × 10^−8^), 40–49 vs. 50–64 (*p* = 0.004) and 40–49 vs. 65–81 (*p* = 0.03)], glycerol-3-phosphate [<40 vs. 50–64 (*p* = 0.04)] and glyceric acid [<40 vs. 40–49 (*p* = 0.006), <40 vs. 50–64 (*p* = 0.0002), <40 vs. 65–81 (*p* = 0.005)] were significant using a critical *p*-value of 0.05. Methionine sulfoxide, also present in greater amounts in the serum of women [*F*(1,901) = 20.3, *p* = 7.7 × 10^−6^], is an oxidation product of methionine and is considered to be a marker of oxidative stress (Bachi et al. 2013) (Fig. 4). Other gender-specific changes in the metabolome as a function of age, BMI and BP were also observed and are discussed below.

### Age

**Fig. 5:**
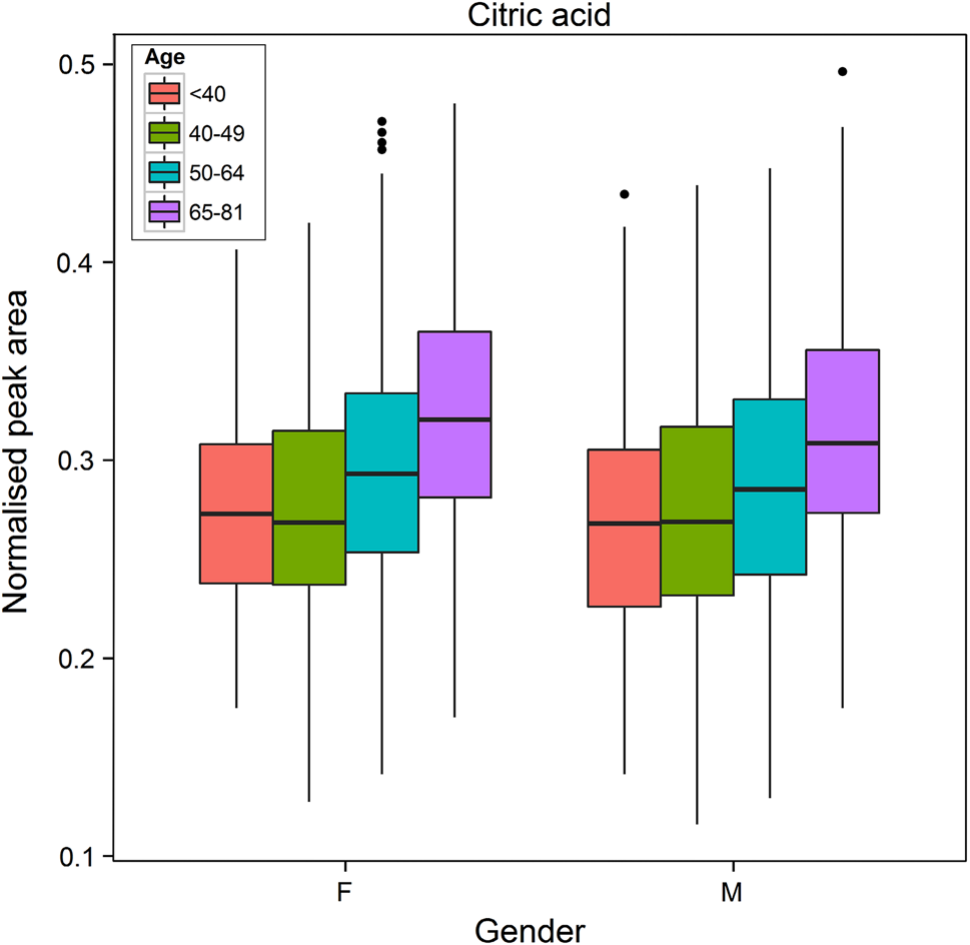
A boxplot showing the distribution of citric acid for males and females across different age categories. For *each box*, the *central line* is the median, the edges of the *box* are the upper and lower quartiles, the whiskers extend the box by a further ±1.5 × interquartile range (IQR), and outliers are plotted as individual points (>1.5 × IQR). Data were analysed using 2-way ANOVA. There was a significant difference between males and females (*F*(1,779) = 79.8, *p* = 3.1 × 10^−18^). There was no significant difference between age categories and no significant interaction between gender and age categories

We assessed age-related changes through the comparison of all subjects below the age of 50 years with all subjects older than 64 years. Two-way ANOVA was performed using Gender and Age (two categories: <50 years, and >64 years) as the main effects. Different classes of metabolites showed changes related to age, with some changes not being gender-related and others being specific to one gender. For example, citric acid showed a general increase with age for both males and females [*F*(1,779) = 79.8, *p* = 3.1 × 10^−18^] and therefore is probably not thus a biomarker for pancreatic cancer (Bathe et al. 2011); visually the rate of increase was greater in females than in males (Fig. 5). Citrate has previously been shown to be related to age, along with other metabolites also observed in our study. These include serine [*F*(1,755) = 6.5, *p* = 0.011], phosphate, aspartate, erythritol/threitol [*F*(1,743) = 171.0, *p* = 2.6 × 10^−35^], caffeine [*F*(1,565) = 8.8, *p* = 0.0032], hexadecenoic acid, glycerol-3-phosphate, histidine, tryptophan [*F*(1,778) = 39.1, *p* = 0.0007], tyrosine [*F*(1,788) = 39.1, *p* = 6.8 × 10^−10^] and threonine [*F*(1,778) = 3.9, *p* = 0.05] (Lawton et al. 2008; Menni et al. 2013). There was a significant difference between gender categories for serine [*F*(1,755) = 7.4, *p* = 0.007], erythritol/threitol [*F*(1,743) = 10.5, *p* = 0.001], caffeine [*F*(1,565) = 24.3, *p* = 1.1 × 10^−6^] and tryptophan [*F*(1,778) = 55.4, *p* = 2.6 × 10^−13^]. There was also a significant interaction between gender and age categories for caffeine [*F*(1,565) = 17.6, *p* = 3.2 × 10^−5^].

**Fig. 6:**
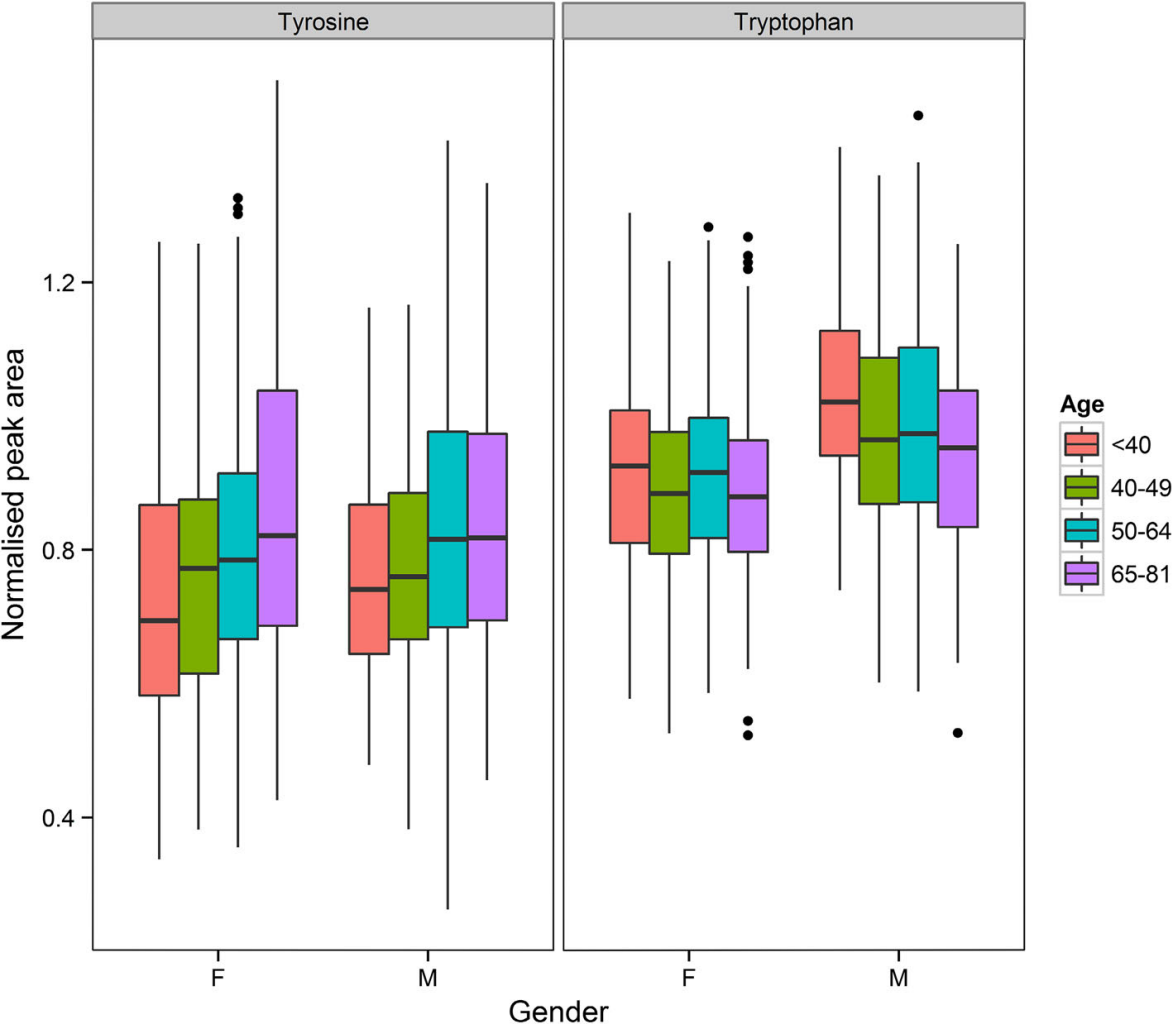
A boxplot showing the distribution of tyrosine and tryptophan for males and females across different age categories. For *each box*, the *central line* is the median, the edges of the *box* are the upper and lower quartiles, the whiskers extend the *box* by a further ±1.5 × interquartile range (IQR), and outliers are plotted as individual points (>1.5 × IQR). Data were analysed using 2-way ANOVA. There was a significant difference across age categories (<50 years vs. >64 years) for tryptophan [*F*(1,778) = 11.7, *p* = 0.0007] and tyrosine [*F*(1,788) = 39.1, *p* = 6.8 × 10^−10^]. There was a significant difference across gender categories for tryptophan [*F*(1,788) = 55.4, *p* = 2.6 × 10^−13^]. There was no significant interaction between gender and age categories for tryptophan or tyrosine

Age-related changes in amino acids were also observed. These changes included tryptophan [*F*(1,778) = 11.7, *p* = 0.0007]; also showed a significant difference between gender categories [*F*(1,778) = 55.4, *p* = 2.6 × 10^−13^] which decreases with age and tyrosine [*F*(1,788) = 39.1, *p* = 6.8 × 10^−10^] which increases with age (as shown in Fig. 6), threonine and serine which both decreased with age and methionine and cysteine [*F*(1,785) = 16.0, *p* = 7.1 × 10^−5^] which also both decreased with age. Cysteine also showed a significant difference between gender categories [*F*(1,785) = 12.9, *p* = 0.0003] and showed a significant interaction between gender and age categories [*F*(1,785) = 4.8, *p* = 0.03]. Vitamin D metabolites also show decreases with age in both males and females, and have been related to the onset of the metabolic syndrome [e.g. Lee et al. (2009), Lu et al. (2009)] and this observation might argue for the benefits of vitamin supplementation in older people. For example, 24-Hydroxygeminivitamin D_3_ showed a difference between age categories [*F*(1,703) = 52.2, *p* = 1.3 × 10^−12^], gender categories [*F*(1,703) = 36.8, *p* = 2.2 × 10^−9^] and a significant interaction between age and gender categories [*F*(1,703) = 5.7, *p* = 0.02]. Different fatty acids showed either increases or decreases with age (e.g. octadecadienoic acid increased with age [*F*(1,763) = 8.6, *p* = 0.003]), but no correlation between age and carbon number, nor degree of saturation, was observed for fatty acids. Erythritol and/or threitol showed an increase (as shown above) with age as did inositol [*F*(1,779) = 151.8, *p* = 5.5 × 10^−32^], which also showed a significant interaction between age and gender categories [*F*(1,779) = 11.3, *p* = 0.0008]. These two changes are consistent with the age-dependent increases in classes of carbohydrates that underpin diabetic complications (Brownlee 2001).

### BMI

While gender and age are independent variables, the body mass index (BMI) is not (although is taken as such for the purposes of this study where one class is BMI <25 and the other class is BMI >30). Nonetheless, with obesity becoming a growing problem in developed and developing countries, even in children (Friend et al. 2013), the measurement of BMI and its relationship to the serum metabolome has become of increasing importance. As is well known, increased BMI is correlated to increases in body fat, greater risk of insulin resistance and metabolic disorders including diabetes and cardiovascular diseases [e.g. Pradhan (2007)]. It should be remembered that BMI is linked to excess weight and the associated risk of insulin resistance and metabolic disorders. BMI is not directly correlated to adiposity as a higher BMI can be related to excess bone, muscle or fat and does not take into account the distribution of the latter and its influence on metabolic diseases. However, BMI provides a readily available surrogate measure of overall body fatness in large-scale studies and was therefore chosen as an appropriate surrogate marker in this study. Two-way ANOVA was performed using BMI (<25 vs. >30) and gender as the main effects.

In this study a range of amino acids showed either an increase (cysteine [*F*(1,690) = 18.8, *p* = 1.6 × 10^−5^], cystine [*F*(1,686) = 16.9, *p* = 4.4 × 10^−5^], glutamine, tyrosine [*F*(1,695) = 62.6, *p* = 9.9 × 10^−15^], phenylalanine [*F*(1,687) = 28.4, *p* = 1.4 × 10^−7^] and valine [*F*(1,685) = 32.0, *p* = 2.2 × 10^−8^]) or decrease (asparagine [*F*(1,687) = 12.8, *p* = 0.0004], histidine, serine [*F*(1,670) = 4.1, *p* = 0.04] and phosphoserine [*F*(1,498) = 29.6, *p* = 8.3 × 10^−8^]) in relative amounts as BMI increased in one or both genders. Cysteine [*F*(1,690) = 11.6, *p* = 0.0007], valine [*F*(1,685) = 53.9, *p* = 6.0 × 10^−13^], serine [*F*(1,670) = 6.9, *p* = 0.009] and phosphoserine [*F*(1,498) = 6.1, *p* = 0.01] also showed a significant difference between gender categories and there was a significant interaction between gender and BMI categories for tyrosine [*F*(1,695) = 4.3, *p* = 0.04] and phosphoserine [*F*(1,498) = 5.7, *p* = 0.02]. Valine, tyrosine and phenylalanine have been strongly linked as early makers of insulin resistance and markers of risk for the development of diabetes (Newgard et al. 2009; Wang et al. 2011). Phosphoserine can be associated with cysteine production, serine metabolism or as a byproduct of protein degradation. Short-chain organic acids (including acetate [*F*(1,645) = 38.4, *p* = 1.1 × 10^−9^], 2-aminobutanoic acid [*F*(1,637) = 8.9, *p* = 0.003] and 2-aminomalonic acid [*F*(1,642) = 57.2, *p* = 1.4 × 10^−13^]) showed a decrease in relative concentration with increasing BMI. 2-Aminomalonic acid also showed a significant difference between gender categories [*F*(1,642) = 34.3, *p* = 7.4 × 10^−9^]. Four diacylglycerides show a decrease as BMI increased, for example, DG(44:6) showed a statistically significant difference applying 2-way ANOVA [*F*(1,489) = 57.0, *p* = 2.1 × 10^−13^] and also showed a significant difference between gender categories [*F*(1,489) = 143.5, *p* = 3.6 × 10^−29^]. Five sphingolipids show a decrease as BMI increased, for example, SM(d18:1/24:1) showed a statistically significant difference applying 2-way ANOVA [*F*(1,518) = 36.0, *p* = 3.8 × 10^−9^] and also showed a significant difference between gender categories [*F*(1,518) = 88.5, *p* = 1.6 × 10^−19^]. Four lyso-glycerophospholipids show a decrease as BMI increased, for example, lysoPC(18:2) showed a statistically significant difference applying 2-way ANOVA [*F*(1,693) = 88.4, *p* = 7.6 × 10^−20^] and also showed a significant difference between gender categories [*F*(1,693) = 27.1, *p* = 2.6 × 10^−7^]. Three fatty acids show a decrease as BMI increased, for example, dodecanoic acid showed a statistically significant difference applying 2-way ANOVA [*F*(1,658) = 20.4, *p* = 7.4 × 10^−6^] and also showed a significant difference between gender categories [*F*(1,658) = 34.9, *p* = 5.7 × 10^−9^]. Citrate and fructoselysine-3-phosphate showed female-specific decreases as a function of BMI. The latter is observed in increased concentrations in tissue and biofluids of diabetic subjects as an Advanced-Glycation Endproduct (AGE) (Delpierre and Van Schaftingen 2003). Glycerol [*F*(1,655) = 43.9, *p* = 7.1 × 10^−11^] and glycerol-3-phosphate showed male-specific increases in amounts (2-way ANOVA results for comparison of gender for glycerol was *F*(1,655) = 91.2, *p* = 2.6 × 10^−20^). Glutamine and glutamate showed an increase and a decrease respectively and threonine showed a decrease as BMI increased. Correlation analysis showed that diglycerides, glycerophosphocholines, sphingomylenins, tyrosine, tyrosyl-arginine and urate also correlated with BMI (Supplementary Fig. 5).

### Blood pressure

Elevated blood pressure (BP) is associated with an increased risk of cardiovascular diseases [e.g. He and Whelton (1999)]. In the UK, up to 38 % of the population is considered hypertensive at one stage or another of their lives, with a greater prevalence of high blood pressure in men. Here we found that a range of metabolic classes in serum were altered in relation to increasing blood pressure when comparing normal blood pressure (systolic = 90–120 mmHg) versus hypertension (systolic >140 mmHg). Two-way ANOVA was performed using Blood Pressure (Normal; Hypertension) and Gender as the main effects.

Methionine sulfoxide was negatively correlated with BP, in both males and females, and methionine showed an increase in relative concentration with blood pressure. One interpretation is that reactive oxygen species that do not oxidize methionine may damage other tissues, leading to a range of disorders (Kell 2009).

Multiple amino acids showed changes including a decrease in cysteine [F(1,589) = 11.5, *p* = 0.0007] and lysine in both males and females whilst other changes were gender specific (e.g., decreased alanine [F(1,567) = 13.3, *p* = 0.0003] and increased tryptophan in males only and increased histidine and decreased threonine in females only). Cysteine also showed a significant difference between gender categories [F(1,589) = 10.2, *p* = 0.001] and there was a significant interaction between gender and BP categories for cysteine [F(1,589) = 8.6, *p* = 0.003]. Lactate relative concentrations were increased in both genders [F(1,587) = 9.3, *p* = 0.002] whilst acetate [*F*(1,543) = 10.1, *p* = 0.002] decreased in females only. Citrulline increased (*F*(1,592) = 5.7, *p* = 0.02) and showed a significant difference between gender categories [*F*(1,592) = 3.9, *p* = 0.05] across both sexes as BP increased, while erythritol/threitol [*F*(1,554) = 10.1, *p* = 0.002] showed a interaction between gender and BP which was statistically significant [*F*(1,554) = 5.8, *p* = 0.02], and erythronic acid/threonic acid decreased in both genders. Glyceric acid and glycerol-3-phosphate both increased and sucrose decreased in both males and females. Other changes included decreases in indole-acetate [*F*(1,561) = 9.7, *p* = 0.002] in males only. Correlation analysis showed links to elevated BP to urate, triacylglycerides, dipeptides, glycerophosphocholines and 4-hydroxyphenyllactic acid (Supplementary Fig. 5).

### Smoking

Smoking is an important risk factor in cancer and cardiovascular diseases; the metabolic disturbances associated with smoking can have important roles in the onset and progression of these diseases. Two-way ANOVA was performed using smoking (non-smoker, ex-smoker, smoker) and gender as the main effects. Correlation analysis showed links between smoking status and salicylic acid, assumedly derived from aspirin and the lifestyle influences on the metabolic phenotype. Smoking was also correlated with the two aromatic amino acids tyrosine ([F(2,796) = 3.7, *p* = 0.02], Tukey post hoc tests showed that, independent of gender, comparisons of smoking categories were statistically significant for smokers vs. non-smokers (*p* = 0.02)) and tryptophan (elevated in smokers). Tryptophan has been associated with smoking initiation and nicotine dependence previously (Wang and Li 2010) and our data show decreases in the metabolically related indole-acetate and indole-propionate ([F(2,757) = 1.4, *p* = 1.3 × 10^−5^]; indole propionate also showed a significant difference between gender categories [F(1,757) = 4.7, *p* = 0.03] and there was a significant interaction between gender and BP categories for indole-propionate [F(2,757) = 6.2, *p* = 0.002]; Tukey post hoc tests showed that, independent of gender, comparisons of smoking categories were statistically significant for smokers vs. non-smokers (*p* = 7.7 × 10^−6^) and smokers vs ex-smokers (*p* = 0.04)). Statistical analysis also showed decreases in other amino acids including aspartate, histidine and lysine in smokers. Glycerol ([*F*(2,759) = 3.3, *p* = 0.04]; glycerol also showed a significant difference between gender categories [*F*(1,759) = 40.8, *p* = 2.9 × 10^−10^]; Tukey post hoc tests showed that, independent of gender, comparisons of smoking categories were statistically significant for smokers vs. non-smokers (*p* = 0.03)) and glycerol-3-phosphate were decreased in smokers as were a number of fatty acids (for example, octadecenoic acid [*F*(2,759) = 3.3, *p* = 0.04]; Tukey post hoc tests showed that, independent of gender, comparisons of smoking categories were statistically significant for non-smokers vs. ex-smokers (*p* = 0.02)). Lactate [*F*(2,778) = 3.5, *p* = 0.03; Tukey post hoc tests showed that, independent of gender, comparisons of smoking categories were statistically significant for non-smokers vs. smokers (*p* = 0.03)] and citrate [*F*(2,800) = 3.9, *p* = 0.02; Tukey post hoc tests showed that, independent of gender, comparisons of smoking categories were statistically significant for non-smokers vs. smokers (*p* = 0.01)] are also decreased in smokers as is inositol [*F*(2,784) = 15.7, *p* = 2.0 × 10^−7^; Tukey post hoc tests showed that, independent of gender, comparisons of smoking categories were statistically significant for non-smokers vs. smokers (*p* = 9.2 × 10^−8^) and for ex-smokers vs. smokers (*p* = 0.0006)]. Biotin was decreased in smokers [*F*(2,814) = 20.0, *p* = 3.2 × 10^−9^; Tukey post hoc tests showed that, independent of gender, comparisons of smoking categories were statistically significant for non-smokers vs. smokers (*p* = 1.3 × 10^−9^) and for ex-smokers vs. smokers (*p* = 0.001)] and this has been shown previously in women (Sealey et al. 2004). Finally caffeine is present at lower relative concentrations in smokers [*F*(2,655) = 8.1, *p* = 0.0003; also showed a significant difference between gender categories [*F*(1,655) = 32.5, *p* = 1.8 × 10^−8^]; Tukey post hoc tests showed that, independent of gender, comparisons of smoking categories were statistically significant for smokers vs. non-smokers (*p* = 0.001) and smokers vs. ex-smokers (*p* = 0.0006)] which is unexpected as there is a logical lifestyle link between coffee drinkers and smokers; however this may show a change in rates of caffeine metabolism in smokers.

### Correlations between clinical chemistry and metabolic profiling data

**Fig. 7:**
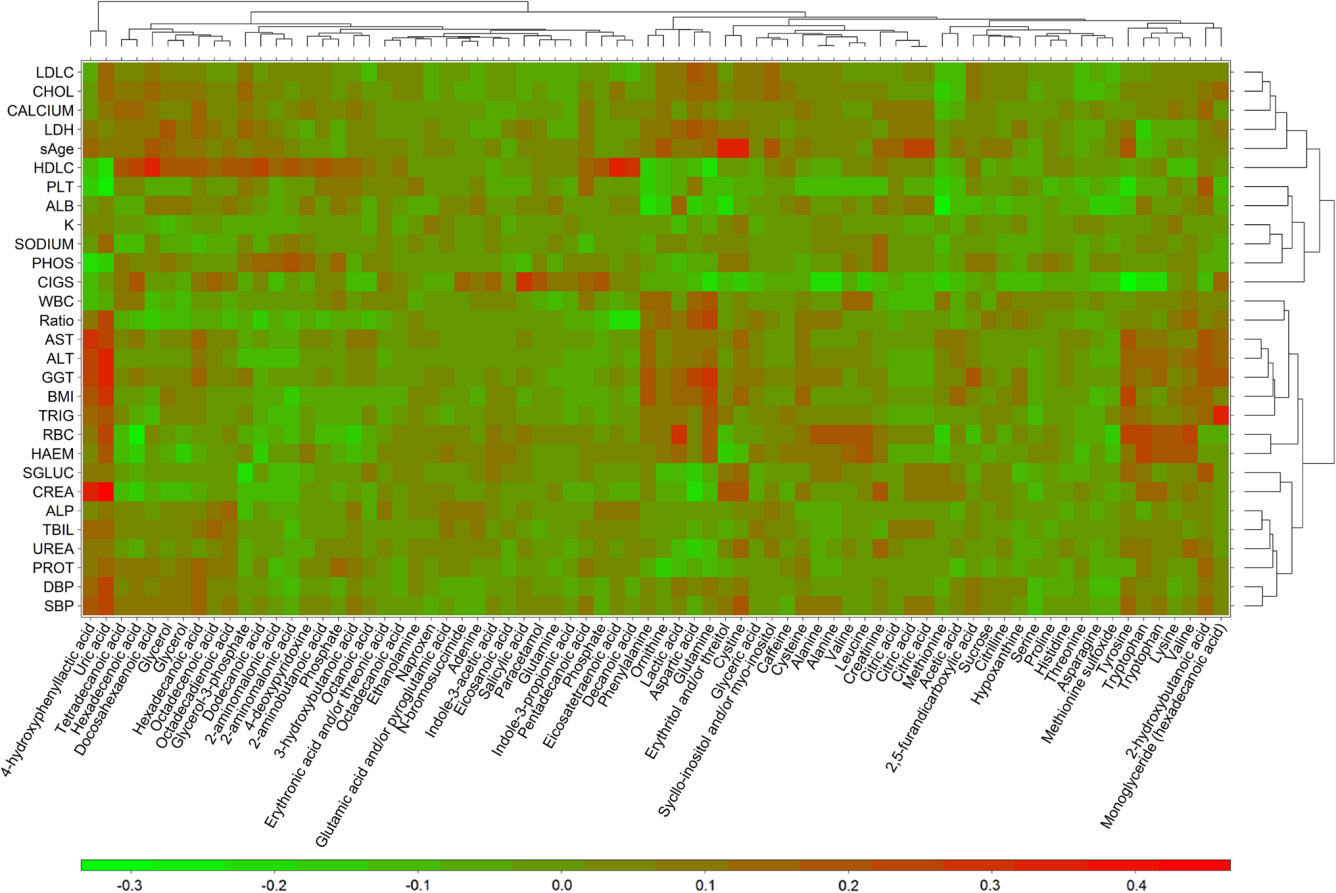
Heatmap with dendrogram of Pearson’s correlation analysis between metabolites detected by GC–MS and clinical chemistry data. The arrangement of the clusters are produced by hierarchical clustering on both metabolites and clinical chemistry data. The *lower bar* represents the *colour code* of coefficients from pairwise Pearson’s correlations between GC–MS data and the clinical chemistry data (Color figure online)

In addition to metabolite profiling, each sample was also subjected to a panel of conventional clinical chemistry assays. This was to enable positive and negative correlations (if any) to these standard clinical diagnostics and the broader metabolic phenotypes to be determined. This ability to anchor newer methods of volunteer/patient phenotyping, in this case metabotyping, with currently used “best practice” represents an important step towards obtaining wider acceptance of the utility of the metabolite profiling approach. The results of this for the correlation of clinical chemistry with GC–MS analysis is illustrated in Fig. 7 (UPLC–MS correlation in Supplementary Fig. 5).

An obvious area where such correlations would be expected is across lipid (and particularly cholesterol) metabolism. As might be expected correlations emerged from the metabotypes determined here between total cholesterol concentrations in serum and the amounts of monoglycerides and diglycerides present. There were also positive correlations between circulating high density lipoprotein cholesterol (HDLC) and relative concentrations of fatty acids, diglycerides, phosphotidylcholines, sphingomylenins and triglycerides, although we were unable to find any correlations for the low density lipoprotein (LDLC). Triglycerides as determined by standard clinical chemistry assays were associated with raised di- and monoglycerides, phosphatidylcholines, sphingomylenins and urate. As discussed above, there was also a correlation of diastolic blood pressure with urate, triglycerides and phosphatidylcholines.

Another set of interesting correlations relating to organ function was seen when some of the clinical markers for liver function were examined. For example, amongst a range of other correlations, both AST and ALT were associated with relative concentrations of urate and 4-hydroxyphenyllactic acid. ALT, in addition, also covaried with acylglycerides and the PC/PE ratio. As observed above, systolic blood pressure (SBP) was also associated with 4-hydroxyphenyllactic acid. Another liver enzyme, GGT varied with diglycerides, glycerophosphocholines, urate, tyrosyl-arginine, aspartate and glutamate whilst no correlations were seen for LDH. In the case of renal function creatinine and urea concentrations were both associated with circulating dipeptides and hexanoylglycine, with creatinine also covarying with phosphatidylcholines, sphingomylenins, urate, erythritol/threitol and triglycerides. Correlations for many other clinical chemistry markers, for e.g., serum glucose, with circulating metabolites were also found.

### Concluding statement and future roles for The Husermet Project

The importance of the Husermet project is that it has developed the tools and resources to collect and provide metabolic profiles based on chromatography-mass spectrometry for a large human population (Dunn et al. 2011). This is a vital prerequisite to well-powered studies that can complement the large-scale but necessarily qualitative studies of genome sequence variation now appearing. Here we describe how these have been applied to profile a sample of the ‘normal’ UK population and for these 1,200 healthy individuals define biologically important metabolic changes associated with age and gender as well to link metabolic changes with disease risk factors including BMI, blood pressure and smoking. It was noteworthy that a significant number of metabolites known to be associated with insulin resistance and the metabolic syndrome did indeed increase with age, indicating the great dangers of a diabesity epidemic in the UK. Additionally, we have correlated metabolic variations with clinical chemistry measurements to indicate metabolic disturbances associated with differences in these variables. ‘Omics’ measurements are normally hypothesis-generating rather than hypothesis testing (Kell and Oliver 2004), although it is always gratifying to be able to reproduce known and published data, as many examples illustrated above have done.

Most importantly, the Husermet protocol has developed a dataset made publicly available through MetaboLights (Haug et al. 2013) so this large resource can be applied as required by the scientific community. An obvious next step is the integration of our data with those for the recently published human metabolic network reconstruction (Swainston et al. 2013) and the other small molecules with which it interacts (Kell 2013; Kell et al. 2013).

## Materials and methods

### Ethics statement

Written informed consent was obtained from each study participant and the study conformed to the principles set out in the WMA Declaration of Helsinki and the NIH Belmont report. The study was approved by the Stockport Local Research Ethics Committee.

### Sample collection

Following assessment of suitable plasticwares such that any plasticizers, phthalates etc. were minimal or absent, serum was collected from 1,200 subjects following appropriate ethical approval of the study; informed consent was acquired from all subjects. A range of clinical parameters were acquired (including age, gender, BMI and smoking status). No data related to medication or food intake were collected. Approximately 10 mL of blood was drawn into serum collection tubes (Greiner, Stonehouse, UK) and was allowed to clot on ice at 4 °C for a minimum of 1 h. The serum fraction was separated by centrifugation (2,500×*g*, 4 °C, 15 min) and 500 µL volumes were aliquoted into separate cryovials (Greiner, Stonehouse, UK). Serum was processed and frozen at −80 °C within 6 h of blood collection. All samples were transported to The University of Manchester on dry ice and stored at −80 °C. Samples were analysed within 2 years of sample collection.

### Sample preparation

All samples were prepared according to a SOP as described previously (Dunn et al. 2011) and will not be described in detail here. In summary, serum was allowed to thaw on ice followed by addition of 1,200 μL of methanol and 200 μL of internal standard solution (0.167 mg mL^−1^ malonic acid *d*_2_, succinic acid *d*_4_, glycine *d*_5_, citric acid *d*_4_, d-fructose ^13^C_6_, l-tryptophan *d*_5_, l-lysine *d*_4_, l-alanine *d*_7_, stearic acid *d*_35_, benzoic acid *d*_5_ and octanoic acid *d*_15_) to 400 μL of serum. The sample was vortex mixed and following centrifugation, four 370 μL aliquots were transferred to separate tubes and dried in a centrifugal vacuum evaporator for 18 h. Quality control (QC) samples were prepared applying a pooled serum sample (Sigma-Aldrich; S7023) as described above.

### Data acquisition

Data were acquired on three analytical platforms (UPLC–MS positive and negative ion modes and GC–MS) according to a SOP as described previously (Dunn et al. 2011) and will not be described in detail here. Samples for UPLC–MS analysis were reconstituted in 100 or 200 μL of water for negative and positive ion modes, respectively and analysed applying reversed-phase UPLC–MS (Waters Acquity UPLC coupled to a Waters LCT mass spectrometer) with a 22 (positive ion mode) or 24 (negative ion mode) minute analysis time. 10 QC samples were analysed at the start of each analytical batch to condition the analytical system and a QC sample was analysed every 5th injection. Samples for GC–MS analysis were prepared applying a two-stage chemical derivatisation procedure (oximation followed by trimethylsilylation) and followed by analysis applying an electron ionisation GC-ToF–MS system (Agilent 6890 N GC coupled to a LECO Pegasus III mass spectrometer). For GC-ToF–MS, 5 QC samples were analysed at the start of each analytical batch to condition the analytical system and a QC sample was analysed every 5th injection. Samples from 1,200 subjects were analysed in 10 different analytical experimental batches, with 120 subject samples analysed in each batch and each batch consisting of analysis across a five day period. Two experimental runs consisting of 60 subjects in each run was operated for UPLC–MS and four experimental runs consisting of 30 subjects in each run was operated for GC–MS. Each batch of 120 subjects was prepared such that it contained a near-random selection of subjects according to the traits in which we are interested (viz. age, gender, BMI, blood pressure, smoking); this was to ensure that any failed batch would not compromise the overall study.

### Data pre-processing

Data were pre-processed and integrated according to a SOP as described previously (Dunn et al. 2011). UPLC–MS data were converted from the raw instrument datafile to NetCDF files and subsequently XCMS was applied for peak deconvolution and alignment separately for each analytical batch. Due to the untargeted nature of the UPLC–MS analysis, the number and identity of common peaks detected in each batch differed considerably. Thus, each of the 20 batched XCMS chromatographic peak-area data matrices consisted of *N*_*b*_ metabolite features (where *b* = 1…20; with *N*_*b*_ associated *m*/*z* and retention times) × 85 samples (60 subjects plus 25 integrated QC samples). GC–MS data were deconvolved and matched to a reference database of 259 metabolites applying ChromaTof (Leco) separately for each analytical batch. This produced 20 chromatographic peak-area data matrices of 259 metabolite features (with associated EI-MS spectrum and retention index) × 80 samples (60 subjects plus 20 integrated QC samples). If a given metabolite was not detected in a given batch then the associated matrix element was replaced with a missing value (NaN; not-a-number).

### Quality assurance, signal correction, batch integration and metabolite identification

For both the GC-Tof–MS and UPLC–MS instrumentation, analytical reproducibility had to be assessed robustly to ensure that data were of comparable high quality within and between analytical batches. The use of periodic analysis of a standard, biologically identical, QC sample within and across all batches, and subsequent statistical assessment of individual peak area variation within and between batches is now highly recommended as a standard quality assurance strategy in metabolite profiling (Dunn et al. 2012). Following preliminary studies (for example, Begley et al. 2009) it has been determined that a tolerance of 20 % RSD for UPLC–MS and 30 % RSD for GC-Tof–MS are acceptable guidelines. Peaks that did not meet acceptable quality thresholds were removed prior to further data analysis. For this study each of the 20 batches was assessed individually, and then data for peaks of high quality were matched across batches. Additionally, it has been shown that for both GC-Tof–MS and UPLC–MS instrumentation there is time dependent non-linear peak area attenuation for many detected metabolite features within a given batch (Begley et al. 2009; Zelena et al. 2009). This problem is compounded with the use of multiple batches, where step changes in instrument sensitivity may be expected. As a pre-processing countermeasure against these phenomena each metabolite feature of a given experimental batch, after XCMS deconvolution, was normalised to the QC sample using robust Locally Weighted Scatterplot Smoothing (LOESS) signal correction (QC-RLSC). Here LOESS was performed on the QC data with respect to the order of injection. A cubic spline correction curve for the whole analytical run was then interpolated, to which the total data set for that peak was normalized. Using this procedure any attenuation of peak response over an analytical run (i.e. confounding factor due to injection order) was minimized, whilst robustly avoiding fitting the correction curve to random measurement error. Normalizing to the QC correction curve also allowed simple data concatenation of high-quality metabolite features across multiple batches. Once combined into a single multi-batch data matrix, each metabolite feature was un-normalized using the overall estimation of expected QC peak area (in this case the median peak area across all batches). Comprehensive details of the quality assurance, signal correction, and batch integration have been described previously (Dunn et al. 2011). For this study a total of 259, 7813 and 7914 unique metabolic features were present in the raw data for GC–MS, UPLC–MS+ and UPLC–MS− respectively. After signal correction, quality assurance, and batch integration there were 126, 2181 and 2283 metabolite features available for further statistical analysis. Each of these features was present in a minimum of 80 % of the samples analyzed. Identification and annotation of metabolites was performed as described previously (Dunn et al. 2011). For UPLC–MS data, the accurate measurement of *m*/*z* followed by grouping of different metabolite features based on retention time similarity, response correlation and expected *m*/*z* differences and the matching of the defined molecular formula for each group of features to those present in a revised MMD database was performed (Brown et al. 2011). For UPLC–MS all metabolite identifications are reported as level 2 (metabolite reported) or level 4 (no metabolite reported) according to the recommendations of the Chemical Analysis Group of the Metabolomics Standards Initiative (MSI) (Sumner et al. 2007). For GC–MS, the electron impact (EI) mass spectrum and retention index were compared to either an in-house EI mass spectral library constructed with authentic chemical standards or other available EI mass spectral libraries (NIST05, Golm Metabolome Database (Kopka et al. 2005)). For GC–MS all metabolites are either identified (MSI level 1; if matched to a metabolite in the in-house library which was constructed applying the same analytical conditions), annotated (MSI level 2; mass spectrum matched to NIST05 or Golm Metabolome Database) or unidentified (MSI level 4).

### Data availability

All metabolite data and associated demographic/clinical metadata are available at the publically available metabolomics data repository MetaboLights (http://www.ebi.ac.uk/metabolights/; study identifier MTBLS97).

### Data analysis

All data analysis follows MSI reporting guidelines (Goodacre et al. 2007). The data from each platform was integrated into single data matrix of 1,187 subjects by 4,261 metabolite features. There were a maximum of 20 % missing values for each metabolite feature and missing values were imputed using the mean value for a given metabolite feature for all subjects. Before statistical analysis each metabolite feature was autoscaled (normalized to unit variance). Initially, for each metabolite feature in turn, the distributions of the classification groups in a given clinical hypothesis (Age; Gender; BMI; Blood Pressure; Smoking) were compared using either the non-parametric Mann–Whitney U test, or Kruskal–Wallis test, depending on the number of groups in the comparison. Additionally, 2-way ANOVA was performed to investigate interactions between clinical variables with respect to metabolite relative concentrations. For all reported 2-way ANOVA results data normality (approximate) was checked, and assured, using Q–Q plots (data not shown).

In order to reduce the high dimensional data set down to a manageable, size a consensus feature selection protocol was implemented for each clinical hypothesis. In this protocol three modeling techniques were utilized: (1) Non-parametric univariate hypothesis testing (as described above), (2) Random Forests (RF) (Breiman 2001) and (3) Partial Least Squares Discriminant Analysis (PLS-DA) (Wold et al. 2001). For a given classification problem, and associated data set, each of these modeling techniques provided a ranked list of metabolite features in order of importance. In order to avoid model over-fitting, and possible false discovery, bootstrap resampling was performed for each modeling technique (Efron and Tibshirani 1993). For both classification and feature selection, 100 bootstrap resamplings (with replacement) were made. The resulting ranked lists of features were averaged using the Borda count consensus voting system (Dwork 2001), resulting in a single aggregated ranked list of metabolite importance. The optimal subset of metabolites for each clinical hypothesis was then found from this rank list using forward selection remodeling. Starting with the most important feature, and adding the next important feature one at a time, a series of classification models were built and associated classification accuracy tested (Cho et al. 2004). The optimal number of metabolite features was at the inflection point in the curve of classification accuracy versus the number of features. On average, across all the clinical hypotheses tested, the inflection point was found at 30 metabolite features with accuracy slightly above 75 %, shown in Supplementary Fig. 6. Therefore we used 30 metabolite features found in GC–MS, positive UPLC–MS and negative UPLC–MS for further annotation analysis in this study. To assess the effectiveness of the feature selection, we applied two classifiers, Random Forest (RF) and Support Vector Machines (SVM) (Cristianini and Shawe-Taylor 2000) to discriminate the categorical groups: age (age <50 and age >65), BMI (BMI <25 and BMI >30) and gender (male and female). A bootstrap re-sampling method was employed to evaluate the performances of the two classifiers. The results shown in Supplementary Fig. 7 reveal that the discrimination with feature selection is much better than those without feature selection, especially for both positive and negative LC-MS data sets.

All annotated metabolites were analysed further by Pearson correlation analysis. We applied two correlation analyses, one between identified metabolite pairs and another between identified metabolites and clinical chemistry data. To visualise the correlation results, we used a heatmap of correlation coefficients. We also applied a hierarchical clustering technique to re-order the correlation coefficients in the heatmap, to highlight the relationship between the variables used.

For large-scale studies of the human population sample size is very important and we therefore studied sample size effects in both classification and feature selection. Selecting sample size ranges varying from 50 to 650 (in steps of 50), we again classified three groups on the basis of age, BMI and gender for three analytical platforms by two classifiers, viz. RF and SVM with 100 bootstrap sample sets. Using the same sample size ranges as for classification, the feature selections were performed using three methods: Wilcoxon test, RF and PLS, combined with a bootstrap re-sampling technique. To examine sample size effects for feature selection, we used correlation analysis to validate the consistency of feature selection on the sample subsets with sample size changing from 50 to 650. The correlation analysis was performed on the aggregated full ranking lists obtained from the three feature selections.

## Electronic supplementary material

Below is the link to the electronic supplementary material.

Supplementary material 1 (DOC 8536 kb)

Supplementary material 2 (XLS 395 kb)

Supplementary material 3 (XLS 504 kb)

Supplementary material 4 (XLSX 152 kb)
