# Supplementary material for: Molecular phenotyping of a UK population: defining the human serum metabolome
Source: Metabolomics. 2014 Jul 25;11(1):9–26. doi: 10.1007/s11306-014-0707-1 (PMC4289517; doi:10.1007/s11306-014-0707-1)

**Dunn et al.**

**Molecular phenotyping of a UK population: defining the human serum metabolome**

**SUPPLEMENTARY INFORMATION**

**Supplementary Figure 1** – Linear Discriminant Analysis scores plots for (a) UPLC-MS(+); (b) UPLC-MS(-) and (c) GC-MS showing no separation of data based on the (timing or other property of the) analytical experiment. Numbers refer to the date order of data acquisition for each analytical batch.

(a) UPLC-MS(+)


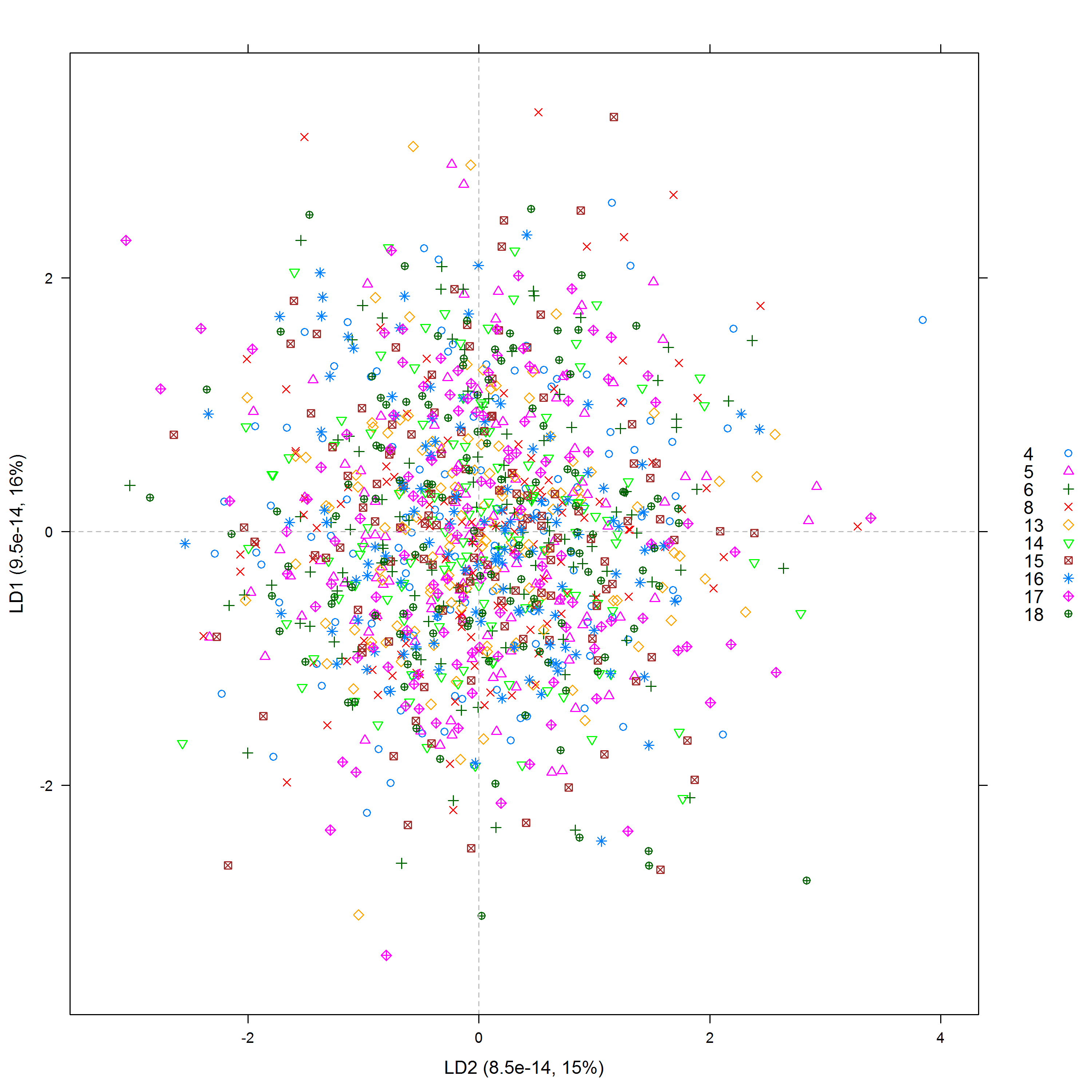


1

2

3

4

5

6

7

8

9

10

(b) UPLC-MS(-)


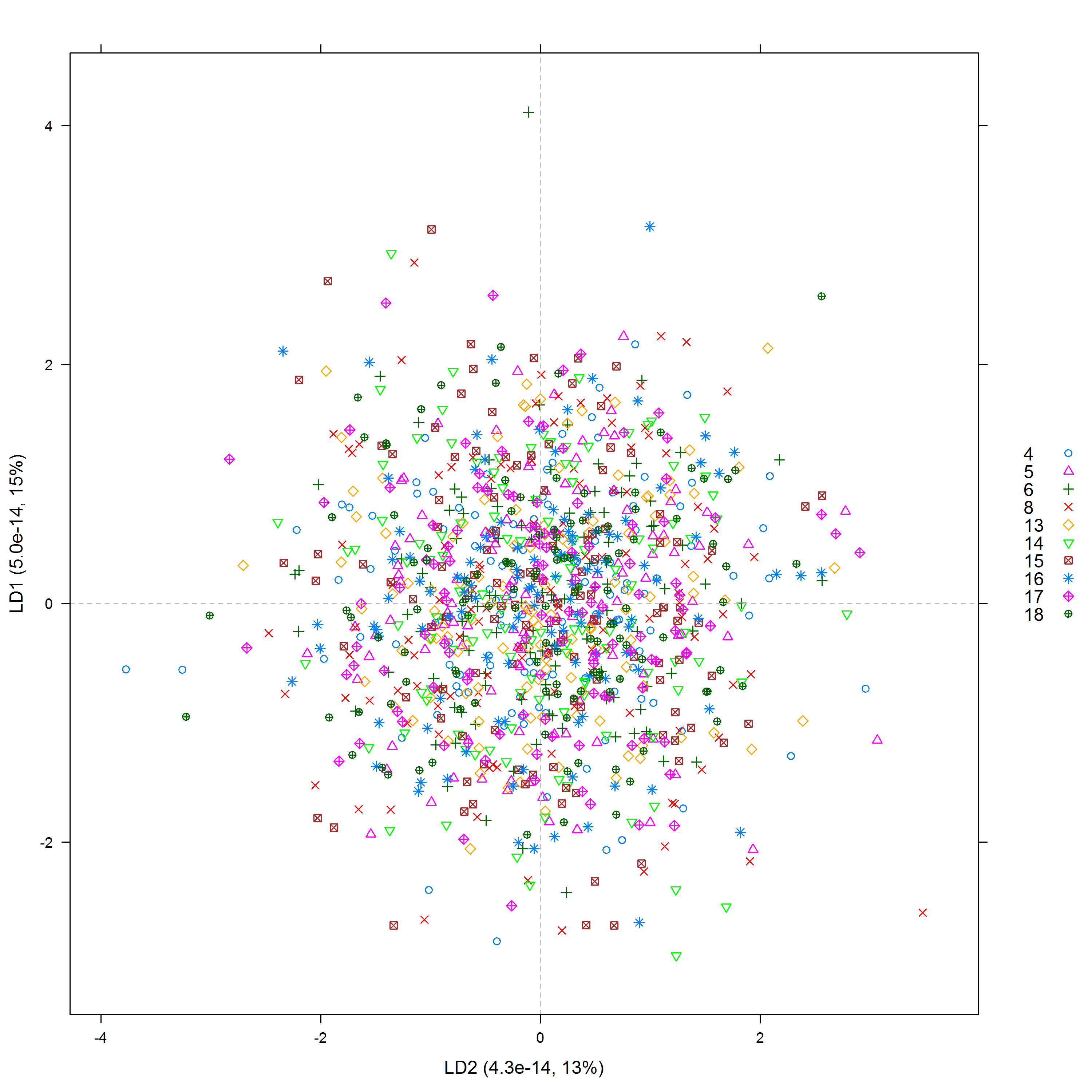


1

2

3

4

5

6

7

8

9

10

(c) GC-MS


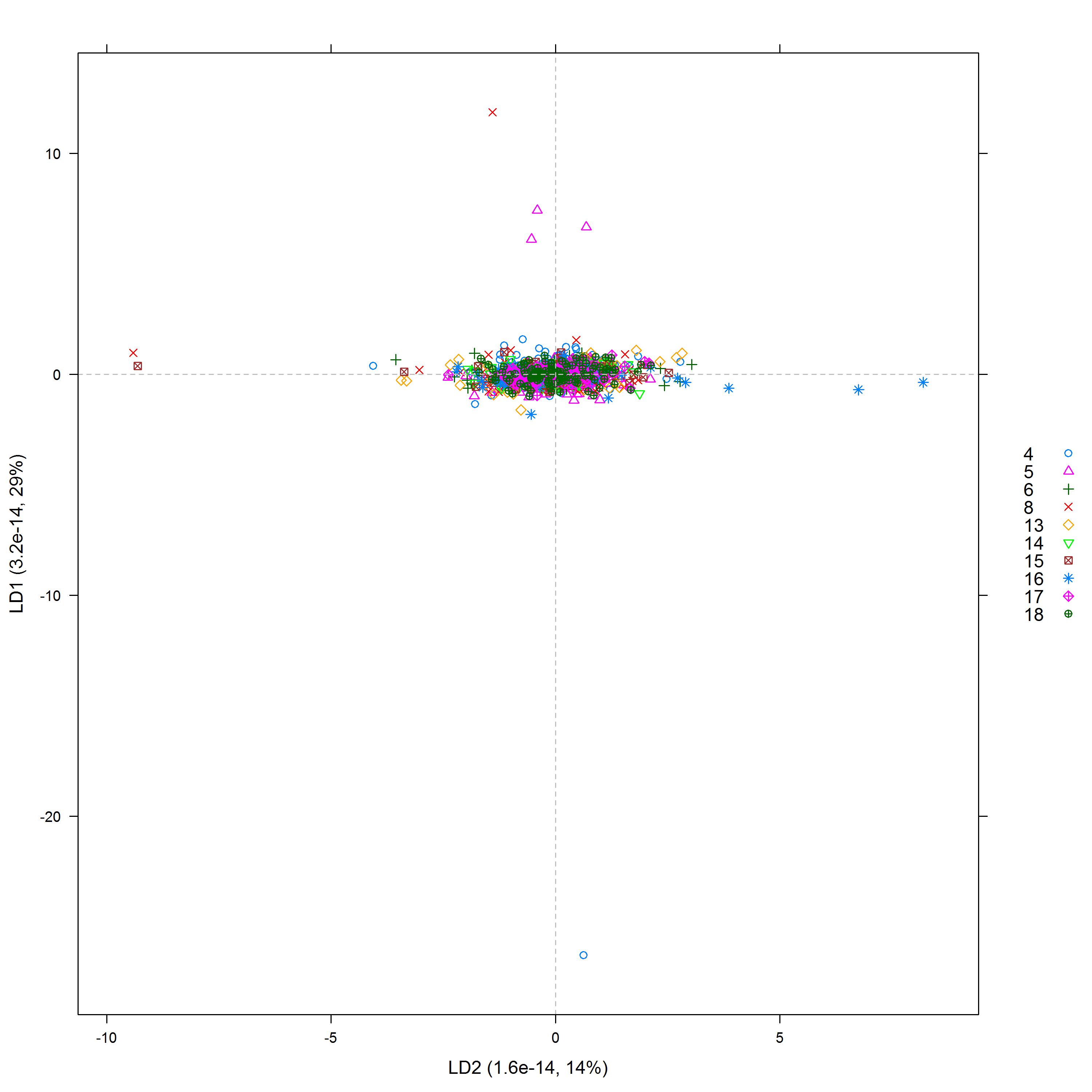


1

2

3

4

5

6

7

8

9

10

**Supplementary Figure 2** –Heatmap with dendrogram of Pearson’s correlation analysis between metabolites detected by (a) UPLC-MS positive and (b) UPLC-MS negative ion modes. The arrangements of the clusters are produced by hierarchical clustering on metabolites.

(a) UPLC-MS(+)


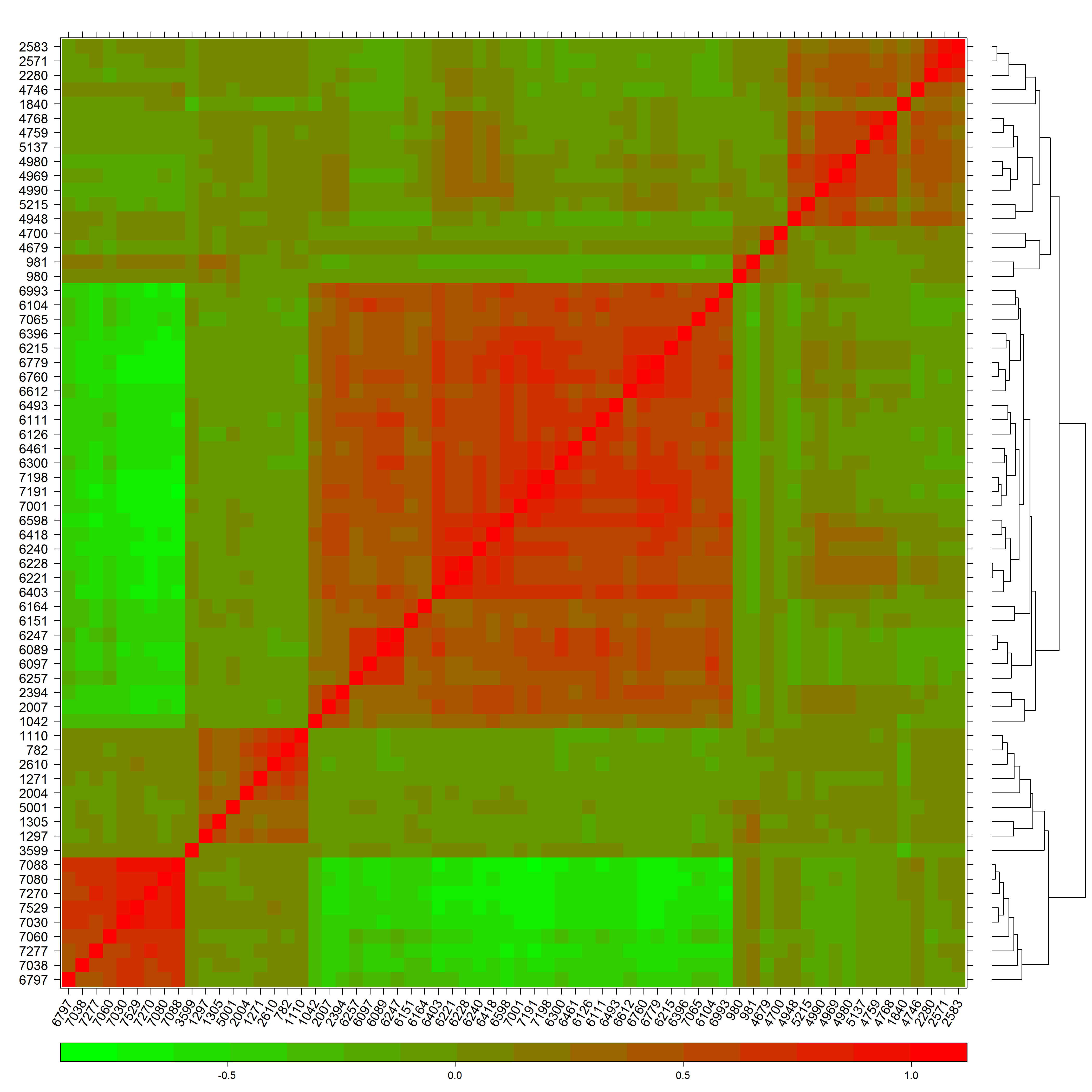


|  |  |
| --- | --- |
| **Idx** | **Metabolite** |
| 782 | Carbohydrate-based metabolite |
| 980 | Dodecenol |
| 981 | L-isoleucyl-L-proline |
| 1042 | beta-Alanyl-L-lysine or gamma-L-Glutamylputrescine |
| 1110 | N-Acetyl-beta-D-galactosamine or N-Acetyl-D-glucosamine or N-Acetyl-D-mannosamine |
| 1271 | 5-Methylthio-5-deoxy-D-ribose 1-phosphate or 5-Methylthio-5-deoxy-D-ribulose 1-phosphate or 2,3-Dioxo-L-gulonate or 2,5-Didehydro-D-gluconate or 2-Dehydro-3-deoxy-D-glucarate or 4,5-Dehydro-D-Glucuronic Acid or 5-Dehydro-4-deoxy-D-glucarate or Citrate or Isocitrate |
| 1297 | L-beta-aspartyl-L-glutamic acid or N-acetyl-seryl-aspartate |
| 1305 | Hexanoylglycine or Isovalerylalanine or Isovalerylsarcosine or N-Acetyl-L-leucine |
| 1840 | Octadecenedienoic acid or octadecapentaenoic acid |
| 2004 | 2-(beta-D-Glucosyl)-sn-glycerol or 3-beta-D-galactosyl-sn-glycerol or 3-beta-D-Galactosyl-sn-glycerol or Galactosylglycerol or Pseudouridine or Uridine or L-prolyl-L-proline |
| 2007 | eicosatrienoic acid or methyl-nonadecatrienoic acid |
| 2280 | MG(16:0) or Isostearic acid or methyl-heptadecanoic acid or dimethyl-hexadecanoic acid or Octadecanoic acid |
| 2394 | 24-Nor-5beta-chol-22-ene-3alpha,12alpha-diol or 24-Nor-5beta-chol-22-ene-3alpha,6alpha-diol or 24-Nor-5beta-chol-22-ene-3alpha,7alpha-diol or 24-Nor-5beta-chol-22-ene-3alpha,7beta-diol |
| 2571 | MG(18:1) or octadecenoic acid or tetramethyl-hexadecenoic acid |
| 2583 | MG(18:1) or octadecenoic acid or tetramethyl-hexadecenoic acid |
| 2610 | Galactosylhydroxylysine |
| 3599 | 5-S-glutathionyl-noradrenochrome hydroquinone |
| 4679 | LysoPS(18:0) or Taurallocholic acid or Tauro-b-muricholic acid or Taurocholate or Taurohyocholate or Tauroursocholic acid |
| 4700 | PE(18:0) |
| 4746 | bacteriohopane-32,33, 34-triol-35-carbamate |
| 4759 | DG(34:3) or DG(32:0) |
| 4768 | DG(33:4) |
| 4948 | DG(36:6) or DG(34:3) or DG(32:0) |
| 4969 | DG(36:5) or DG(34:2) |
| 4980 | DG(36:5) or DG(34:2) |
| 4990 | DG(36:5) or DG(34:2) |
| 5001 | dTDP-3-methyl-4-oxo-2,6-dideoxy-L-glucose or dTDP-4-oxo-3-methyl-2,6-dideoxy-beta-L-glucose or Thymidine-5'-Diphospho-Beta-D-Xylose or dTDP-3,4-dioxo-2,6-dideoxy-D-glucose |
| 5137 | DG(34:1) |
| 5215 | DG(38:6) or DG(36:3) or DG(34:0) |
| 6089 | PC(16:1/dm18:1) or PC(18:2/dm16:0) or PC(O-16:0/18:3) or PC(P-16:0/18:2) or PC(16:0/O-16:0) or PC(O-14:0/18:0) |
| 6097 | PC(16:1/dm18:1) or PC(18:2/dm16:0) or PC(O-16:0/18:3) or PC(P-16:0/18:2) or PC(16:0/O-16:0) or PC(O-14:0/18:0) |
| 6104 | PC(16:0/dm18:1) or PC(16:1/dm18:0) or PC(18:1/dm16:0) or PC(O-16:0/18:2) or PC(O-16:1/18:1) or PC(P-16:0/18:1) or Coenzyme Q10 |
| 6111 | DG(40:2) |
| 6126 | PC(16:0/dm18:0) or PC(18:0/dm16:0) or PC(O-16:0/18:1) or PC(O-18:0/16:1) or PC(O-18:1/16:0) |
| 6151 | PE(20:4/dm18:1) or PE(20:5/dm18:0) or PE(22:5/dm16:0) or PE(18:1/dm18:1) or PE(18:2/dm18:0) or PE(20:2/dm16:0) |
| 6164 | PE(20:3/dm18:1) or PE(20:4/dm18:0) or PE(22:4/dm16:0) or PE(O-16:0/22:5) or PE(O-18:0/20:5) or PE(O-18:1/20:4) or PE(18:0/dm18:1) or PE(18:1/dm18:0) or PE(20:1/dm16:0) or PE(P-18:0/18:1) |
| 6215 | SM(d18:1/20:0) |
| 6221 | PC(34:1) or PE-NMe2(O-16:0/O-16:0) |
| 6228 | PC(34:1) or PE-NMe2(O-16:0/O-16:0) |
| 6240 | DG(42:2) or CE(22:2) or CE(20:1) |
| 6247 | PC(16:1/dm18:1) or PC(18:2/dm16:0) or PC(O-16:0/18:3) or PC(P-16:0/18:2) or PC(16:0/O-16:0) or PC(O-14:0/18:0) |
| 6257 | DG(42:6) or DG(40:3) |
| 6300 | Plastoquinone-9 or PE(20:2/dm18:1) or PE(20:3/dm18:0) or DG(40:0) |
| 6396 | SM(d18:1/22:1) |
| 6403 | PC(36:2) |
| 6418 | PC(36:1) or PE(O-18:0/O-18:0) or PE(O-20:0/O-16:0) |
| 6461 | DG(44:6) |
| 6493 | PE(22:4/dm18:0) or DG(42:1) |
| 6598 | SM(d18:0/22:0) |
| 6612 | SM(d18:1/24:1) |
| 6760 | SM(d18:0/22:0) |
| 6779 | SM(d18:1/24:1) |
| 6797 | (6S)-6-beta-Hydroxy-1,4,5,6-tetrahydronicotinamide-adenine dinucleotide 2'-phosphate |
| 6993 | PC(22:2/dm18:1) or PE(40:0) |
| 7001 | PE(44:7) or 3-Decaprenyl-4-hydroxybenzoic acid |
| 7030 | PC(20:3/dm18:1) or PC(20:4/dm18:0) or PC(22:4/dm16:0) or PC(O-16:0/22:5) or PC(O-18:0/20:5) or PC(O-18:1/20:4) or PC(P-18:0/20:4) or PC(36:3) or PE(22:4/dm18:1) or PE(22:5/dm18:0) |
| 7038 | Ubiquinone-9 or PG(38:3) or PG(36:0) or DG(46:6) |
| 7060 | PE(40:3) or PC(38:7) or PC(36:4) |
| 7065 | PC(22:0/dm18:1)PC(22:1/dm18:0) or PC(24:1/dm16:0) or PE(40:0) |
| 7080 | PC(38:6) or PC(36:3) or CerP(d18:1/26:0) or PC(O-18:1/O-18:1) |
| 7088 | PC(38:6) or PC(36:3) or CerP(d18:1/26:0) or PC(O-18:1/O-18:1) |
| 7191 | SM(d18:0/24:1) or SM(d18:1/24:0) |
| 7198 | SM(d18:0/24:1) or SM(d18:1/24:0) |
| 7270 | Galabiosylceramide (d18:1/16:0) or Lactosylceramide (d18:1/16:0) or PE(42:3) or PC(40:7) or PC(38:4) or CerP(d18:1/26:1) |
| 7277 | SM(d18:1/22:1) |
| 7529 | PC(22:6/dm18:1) |

(b) UPLC-MS(-)


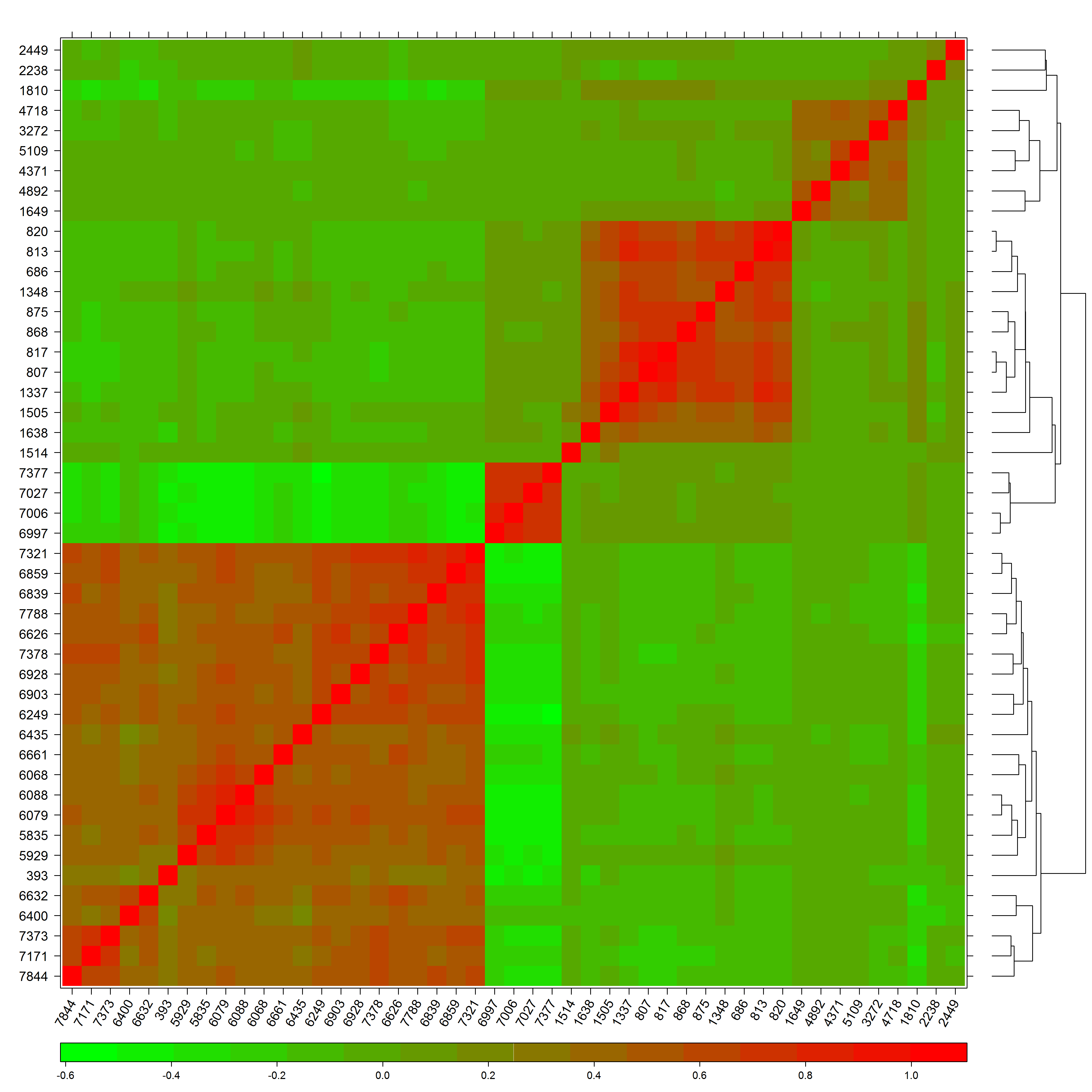


| **Idx** | **Metabolite** |
| --- | --- |
| 393 | Nitro-Cresol or Aminosalicylic acid or Hydroxylaminobenzoate |
| 686 | 3,3-Dimethylglutaric acid or 3-Methyladipic acid |
| 807 | Carbohydrate-based metabolite |
| 813 | Carbohydrate-based metabolite |
| 817 | Carbohydrate-based metabolite |
| 820 | Carbohydrate-based metabolite |
| 868 | Carbohydrate-based metabolite |
| 875 | Carbohydrate-based metabolite |
| 1337 | Allopurinol riboside |
| 1348 | 5-Methyldeoxycytidine |
| 1505 | 3''-Deamino-3''-oxonicotianamine or N-Glucosylnicotinate |
| 1514 | Glycerophosphocholine |
| 1638 | N6,N6-Dimethyladenosine or 1D-1-Guanidino-3-amino-1,3-dideoxy-scyllo-inositol 4-phosphate or 1D-1-Guanidino-3-amino-1,3-dideoxy-scyllo-inositol 6-phosphate |
| 1649 | 4-Hydroxy-3-polyprenylbenzoate or N-Caffeoylputrescine or L-isoleucyl-L-proline or L-leucyl-L-proline |
| 1810 | Uric acid |
| 2238 | L-Tyrosyl-L-arginine |
| 2449 | tetracosadienoic acid or MG(18:1) |
| 3272 | Leukotriene E3 or N-acetylsphingosine 1-phosphate |
| 4371 | 15,16-Dihydrobiliverdin OR Bilirubin |
| 4718 | 26,27-diethyl-1alpha,25-dihydroxy-22-thia-20-epivitamin D3 or 26,27-diethyl-1alpha,25-dihydroxy-22-thiavitamin D3 |
| 4892 | Mesobiliverdin Iv Alpha |
| 5109 | Urobilin |
| 5835 | Ubiquinone-8 or SM(d18:0/16:0) or CE(18:0) |
| 5929 | PC(O-14:0/16:0) |
| 6068 | Ubiquinol 8 or SM(d18:0/18:1) or CE(20:1) |
| 6079 | PE(20:2/dm18:1) or PE(20:3/dm18:0) |
| 6088 | SM(d18:0/18:0) or CE(20:0) |
| 6249 | PE(38:2) or PC(O-16:0/O-18:0) or GalCer(d18:1/18:1) or GluCer(d18:1/18:1) |
| 6400 | DG(46:7) or CE(22:5) |
| 6435 | PE(40:5) or PE(36:0) or PG(36:1) or GalCer(d18:1/20:0) or GluCer(d18:1/20:0) |
| 6626 | PE(40:2) or DG(46:3) |
| 6632 | PC(18:0/dm18:1) or PC(18:1/dm18:0) or PC(18:2/O-18:0) or PC(20:1/dm16:0) or PC(P-18:0/18:1) |
| 6661 | PE(42:5) or PE(40:2) or PE(38:0) or GalCer(d18:1/22:0) or GluCer(d18:1/22:0) |
| 6839 | PC(40:0) |
| 6859 | TG(52:7) or TG(50:4) or PE(42:0) or TG(48:1) or DG(46:0) or docosanyl octacosanoate or dotriacontanyl octadecanoate or octacosanyl docosanoate or tetratriacontanyl hexadecanoate or triacontanyl icosanoate |
| 6903 | SM(d18:0/24:0) or PC(40:4) |
| 6928 | docosanyl octacosanoate or dotriacontanyl octadecanoate or octacosanyl docosanoate or tetratriacontanyl hexadecanoate or triacontanyl icosanoate |
| 6997 | PC(38:2) or PC(36:1) |
| 7006 | PC(42:9) or PG(40:4) or DG(46:4) |
| 7027 | PC(42:8) or SM(d18:1/22:1) or DG(46:3) or TG(48:3) |
| 7171 | PC(44:3) or PC(42:0) or PC(24:0/dm18:0) |
| 7321 | PC(46:7) or PC(44:4) or PC(O-18:0/22:0) |
| 7373 | TG(58:12) or TG(56:9) |
| 7377 | PC(44:8) |
| 7378 | PC(24:0/dm18:1) or PC(24:1/dm18:0) |
| 7788 | PC(44:5) or PC(24:0/dm18:1) |
| 7844 | Galabiosylceramide (d18:1/22:0) or Lactosylceramide (d18:1/22:0) |

**Supplementary Table 1** – Table defining the maximum number of samples in each class in a study of the effect of sample size in terms of the prediction power of classification and the consistency of feature selection applying Random Forests and Support vector Machine. Three separate binary classifications were applied; age, gender and BMI.

| Platform | Age | | BMI | | Gender | |
| --- | --- | --- | --- | --- | --- | --- |
|  | <50 years | >65 years | <25 | >30 | male | female |
| GC-MS | 615 | 182 | 494 | 204 | 478 | 689 |
| UPLC-MS(+) | 631 | 183 | 510 | 206 | 490 | 699 |
| UPLC-MS(-) | 631 | 183 | 509 | 206 | 490 | 698 |

**Supplementary Figure 3 –** Accuracy of classification analysis to assess sample size effects in large-scale studies. The accuracy rate of discrimination with 95% confidence intervals for data acquired applying GC-MS (gc), UPLC-MS negative ion mode (lc_neg) and UPLC-MS positive ion mode (lc_pos) for the three parameters of age (age<50 *vs.* age>65), BMI (BMI<25 *vs.* BMI>30) and gender (male *vs*. female) is shown. The top panel shows results applying a Random Forest (RF) classifier and the bottom panel shows results applying a Support Vector Machine (SVM) classifier. 100 bootstrap sample sets were used for the assessment of classification accuracy.





**Supplementary Figure 4 –** Correlation analysis of feature selection for sample size effects. Three categorical groups, age (age<50 and age>65), bmi(bmi<25 and bmi>30) and gender (male and female) in analytical platforms GC-MS, UPLC-MS(+) and UPLC-MS(-) were used for the study. The aggregated ranking list from three feature selection methods, Wilcoxon test, random forest (RF) and partial least square (PLS) was produced for the each sample size varying from 50 to 650. 100 bootstrap sets were used for the feature selection. The relationship of feature selection among these sample size are evaluated by Pearson’s correlation analysis.


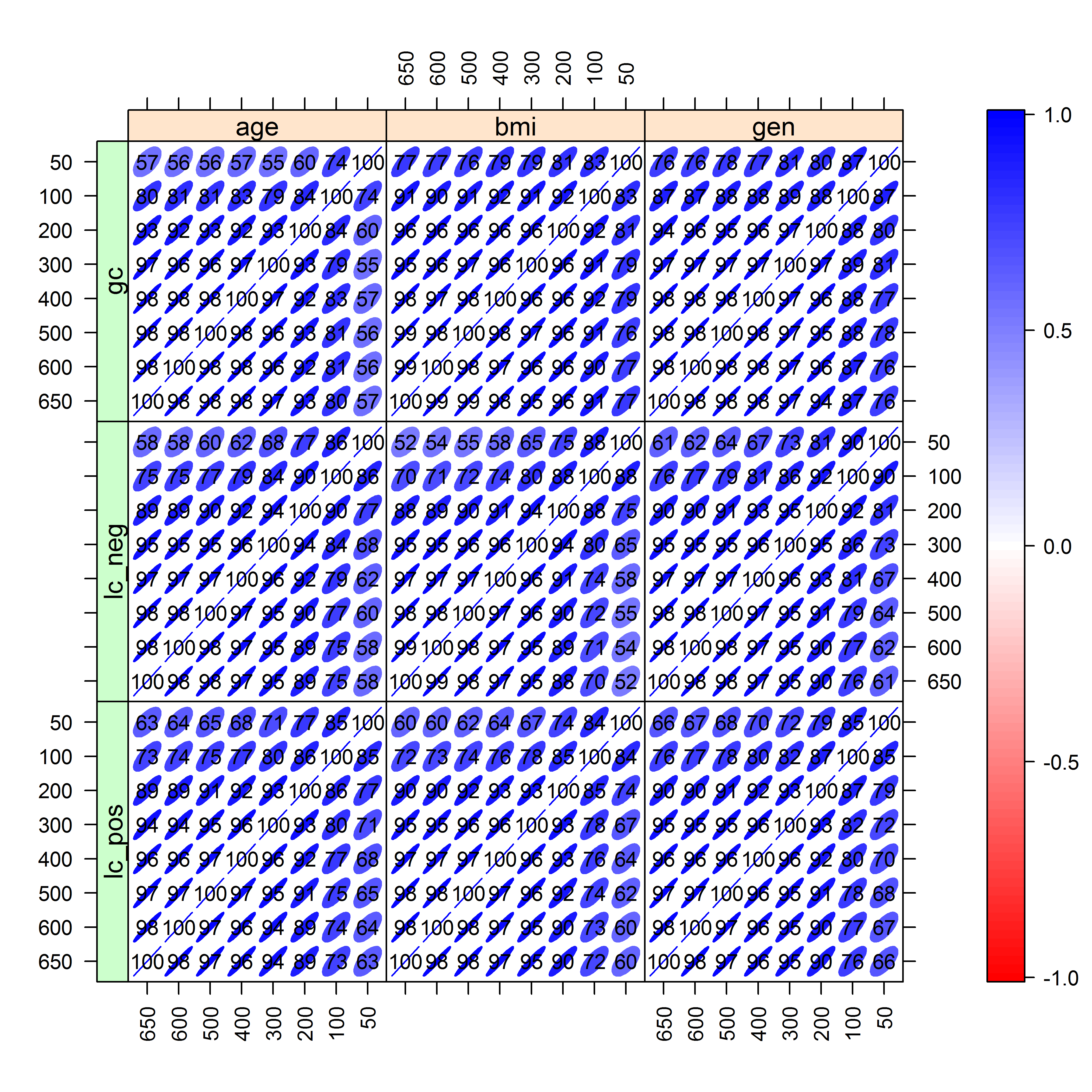


**Supplementary Information 6 –** Results of data analysis performed applying consensus feature selection as described in the methods section and describing observed metabolic changes in serum related to gender, age, BMI, blood pressure and smoking. Results are provided for GC-MS, UPLC-MS(+) and UPLC-MS(-). Results available as a separate Excel file.

**Supplementary Information 7 –** Results of data analysis performed applying two-way analysis of variance (ANOVA) and their post-hoc analysis by Tukey's HSD (“honestly significant difference”) test as described in the methods section and describing observed metabolic changes in serum related to gender, age, BMI, blood pressure and smoking. Results are provided for GC-MS data. Results available as a separate Excel file.

**Supplementary Information 8 –** Results of data analysis performed applying two-way analysis of variance (ANOVA) and their post-hoc analysis by Tukey's HSD (“honestly significant difference”) test as described in the methods section and describing observed metabolic changes in serum related to gender, age, BMI, blood pressure and smoking. Results are provided for UPLC-MS(+) and UPLC-MS(-) data. Results available as a separate Excel file.

**Supplementary Figure 5** - Heatmap with dendrogram of Pearson’s correlation analysis between clinical chemistry data and metabolites detected by (a) UPLC-MS (positive ion mode) and (b) UPLC-MS (negative ion mode). The arrangement of the clusters is produced by hierarchical clustering on both metabolites and clinical chemistry data. The lower bar represents the colour code used for R from pairwise Pearson’s correlations between UPLC-MS data and the clinical chemistry data. Metabolite annotations for each metabolite identifier are shown below each heatmap.

(a)


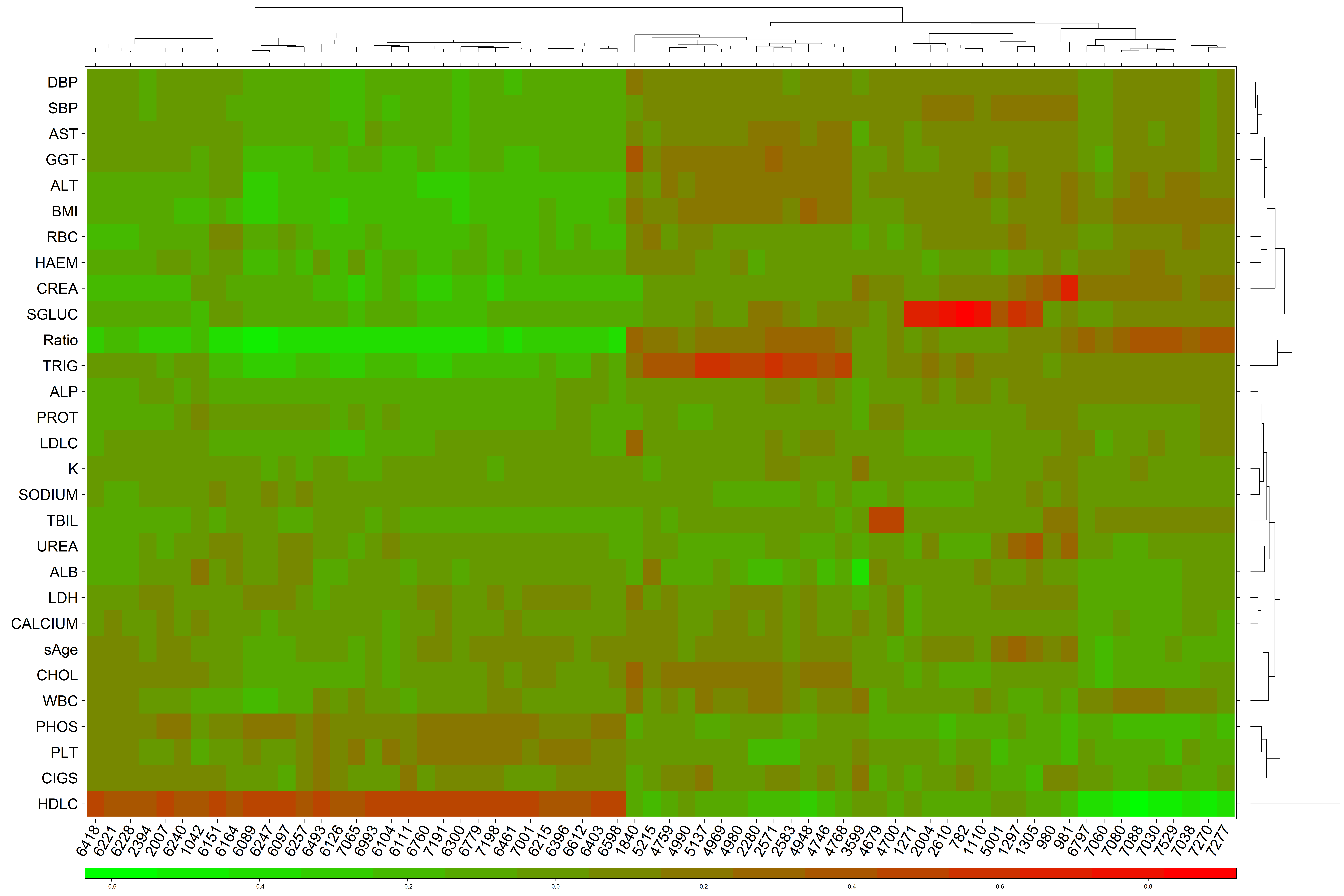


| Idx | Metabolite |
| --- | --- |
| 782 | Carbohydrate-based metabolite |
| 980 | Dodecenol |
| 981 | L-isoleucyl-L-proline |
| 1042 | beta-Alanyl-L-lysine or gamma-L-Glutamylputrescine |
| 1110 | N-Acetyl-beta-D-galactosamine or N-Acetyl-D-glucosamine or N-Acetyl-D-mannosamine |
| 1271 | 5-Methylthio-5-deoxy-D-ribose 1-phosphate or 5-Methylthio-5-deoxy-D-ribulose 1-phosphate or 2,3-Dioxo-L-gulonate or 2,5-Didehydro-D-gluconate or 2-Dehydro-3-deoxy-D-glucarate or 4,5-Dehydro-D-Glucuronic Acid or 5-Dehydro-4-deoxy-D-glucarate or Citrate or Isocitrate |
| 1297 | L-beta-aspartyl-L-glutamic acid or N-acetyl-seryl-aspartate |
| 1305 | Hexanoylglycine or Isovalerylalanine or Isovalerylsarcosine or N-Acetyl-L-leucine |
| 1840 | Octadecenedienoic acid or octadecapentaenoic acid |
| 2004 | 2-(beta-D-Glucosyl)-sn-glycerol or 3-beta-D-galactosyl-sn-glycerol or 3-beta-D-Galactosyl-sn-glycerol or Galactosylglycerol or Pseudouridine or Uridine or L-prolyl-L-proline |
| 2007 | eicosatrienoic acid or methyl-nonadecatrienoic acid |
| 2280 | MG(16:0) or Isostearic acid or methyl-heptadecanoic acid or dimethyl-hexadecanoic acid or Octadecanoic acid |
| 2394 | 24-Nor-5beta-chol-22-ene-3alpha,12alpha-diol or 24-Nor-5beta-chol-22-ene-3alpha,6alpha-diol or 24-Nor-5beta-chol-22-ene-3alpha,7alpha-diol or 24-Nor-5beta-chol-22-ene-3alpha,7beta-diol |
| 2571 | MG(18:1) or octadecenoic acid or tetramethyl-hexadecenoic acid |
| 2583 | MG(18:1) or octadecenoic acid or tetramethyl-hexadecenoic acid |
| 2610 | Galactosylhydroxylysine |
| 3599 | 5-S-glutathionyl-noradrenochrome hydroquinone |
| 4679 | LysoPS(18:0) or Taurallocholic acid or Tauro-b-muricholic acid or Taurocholate or Taurohyocholate or Tauroursocholic acid |
| 4700 | PE(18:0) |
| 4746 | bacteriohopane-32,33, 34-triol-35-carbamate |
| 4759 | DG(34:3) or DG(32:0) |
| 4768 | DG(33:4) |
| 4948 | DG(36:6) or DG(34:3) or DG(32:0) |
| 4969 | DG(36:5) or DG(34:2) |
| 4980 | DG(36:5) or DG(34:2) |
| 4990 | DG(36:5) or DG(34:2) |
| 5001 | dTDP-3-methyl-4-oxo-2,6-dideoxy-L-glucose or dTDP-4-oxo-3-methyl-2,6-dideoxy-beta-L-glucose or Thymidine-5'-Diphospho-Beta-D-Xylose or dTDP-3,4-dioxo-2,6-dideoxy-D-glucose |
| 5137 | DG(34:1) |
| 5215 | DG(38:6) or DG(36:3) or DG(34:0) |
| 6089 | PC(16:1/dm18:1) or PC(18:2/dm16:0) or PC(O-16:0/18:3) or PC(P-16:0/18:2) or PC(16:0/O-16:0) or PC(O-14:0/18:0) |
| 6097 | PC(16:1/dm18:1) or PC(18:2/dm16:0) or PC(O-16:0/18:3) or PC(P-16:0/18:2) or PC(16:0/O-16:0) or PC(O-14:0/18:0) |
| 6104 | PC(16:0/dm18:1) or PC(16:1/dm18:0) or PC(18:1/dm16:0) or PC(O-16:0/18:2) or PC(O-16:1/18:1) or PC(P-16:0/18:1) or Coenzyme Q10 |
| 6111 | DG(40:2) |
| 6126 | PC(16:0/dm18:0) or PC(18:0/dm16:0) or PC(O-16:0/18:1) or PC(O-18:0/16:1) or PC(O-18:1/16:0) |
| 6151 | PE(20:4/dm18:1) or PE(20:5/dm18:0) or PE(22:5/dm16:0) or PE(18:1/dm18:1) or PE(18:2/dm18:0) or PE(20:2/dm16:0) |
| 6164 | PE(20:3/dm18:1) or PE(20:4/dm18:0) or PE(22:4/dm16:0) or PE(O-16:0/22:5) or PE(O-18:0/20:5) or PE(O-18:1/20:4) or PE(18:0/dm18:1) or PE(18:1/dm18:0) or PE(20:1/dm16:0) or PE(P-18:0/18:1) |
| 6215 | SM(d18:1/20:0) |
| 6221 | PC(34:1) or PE-NMe2(O-16:0/O-16:0) |
| 6228 | PC(34:1) or PE-NMe2(O-16:0/O-16:0) |
| 6240 | DG(42:2) or CE(22:2) or CE(20:1) |
| 6247 | PC(16:1/dm18:1) or PC(18:2/dm16:0) or PC(O-16:0/18:3) or PC(P-16:0/18:2) or PC(16:0/O-16:0) or PC(O-14:0/18:0) |
| 6257 | DG(42:6) or DG(40:3) |
| 6300 | Plastoquinone-9 or PE(20:2/dm18:1) or PE(20:3/dm18:0) or DG(40:0) |
| 6396 | SM(d18:1/22:1) |
| 6403 | PC(36:2) |
| 6418 | PC(36:1) or PE(O-18:0/O-18:0) or PE(O-20:0/O-16:0) |
| 6461 | DG(44:6) |
| 6493 | PE(22:4/dm18:0) or DG(42:1) |
| 6598 | SM(d18:0/22:0) |
| 6612 | SM(d18:1/24:1) |
| 6760 | SM(d18:0/22:0) |
| 6779 | SM(d18:1/24:1) |
| 6797 | (6S)-6-beta-Hydroxy-1,4,5,6-tetrahydronicotinamide-adenine dinucleotide 2'-phosphate |
| 6993 | PC(22:2/dm18:1) or PE(40:0) |
| 7001 | PE(44:7) or 3-Decaprenyl-4-hydroxybenzoic acid |
| 7030 | PC(20:3/dm18:1) or PC(20:4/dm18:0) or PC(22:4/dm16:0) or PC(O-16:0/22:5) or PC(O-18:0/20:5) or PC(O-18:1/20:4) or PC(P-18:0/20:4) or PC(36:3) or PE(22:4/dm18:1) or PE(22:5/dm18:0) |
| 7038 | Ubiquinone-9 or PG(38:3) or PG(36:0) or DG(46:6) |
| 7060 | PE(40:3) or PC(38:7) or PC(36:4) |
| 7065 | PC(22:0/dm18:1)PC(22:1/dm18:0) or PC(24:1/dm16:0) or PE(40:0) |
| 7080 | PC(38:6) or PC(36:3) or CerP(d18:1/26:0) or PC(O-18:1/O-18:1) |
| 7088 | PC(38:6) or PC(36:3) or CerP(d18:1/26:0) or PC(O-18:1/O-18:1) |
| 7191 | SM(d18:0/24:1) or SM(d18:1/24:0) |
| 7198 | SM(d18:0/24:1) or SM(d18:1/24:0) |
| 7270 | Galabiosylceramide (d18:1/16:0) or Lactosylceramide (d18:1/16:0) or PE(42:3) or PC(40:7) or PC(38:4) or CerP(d18:1/26:1) |
| 7277 | SM(d18:1/22:1) |
| 7529 | PC(22:6/dm18:1) |

(b)


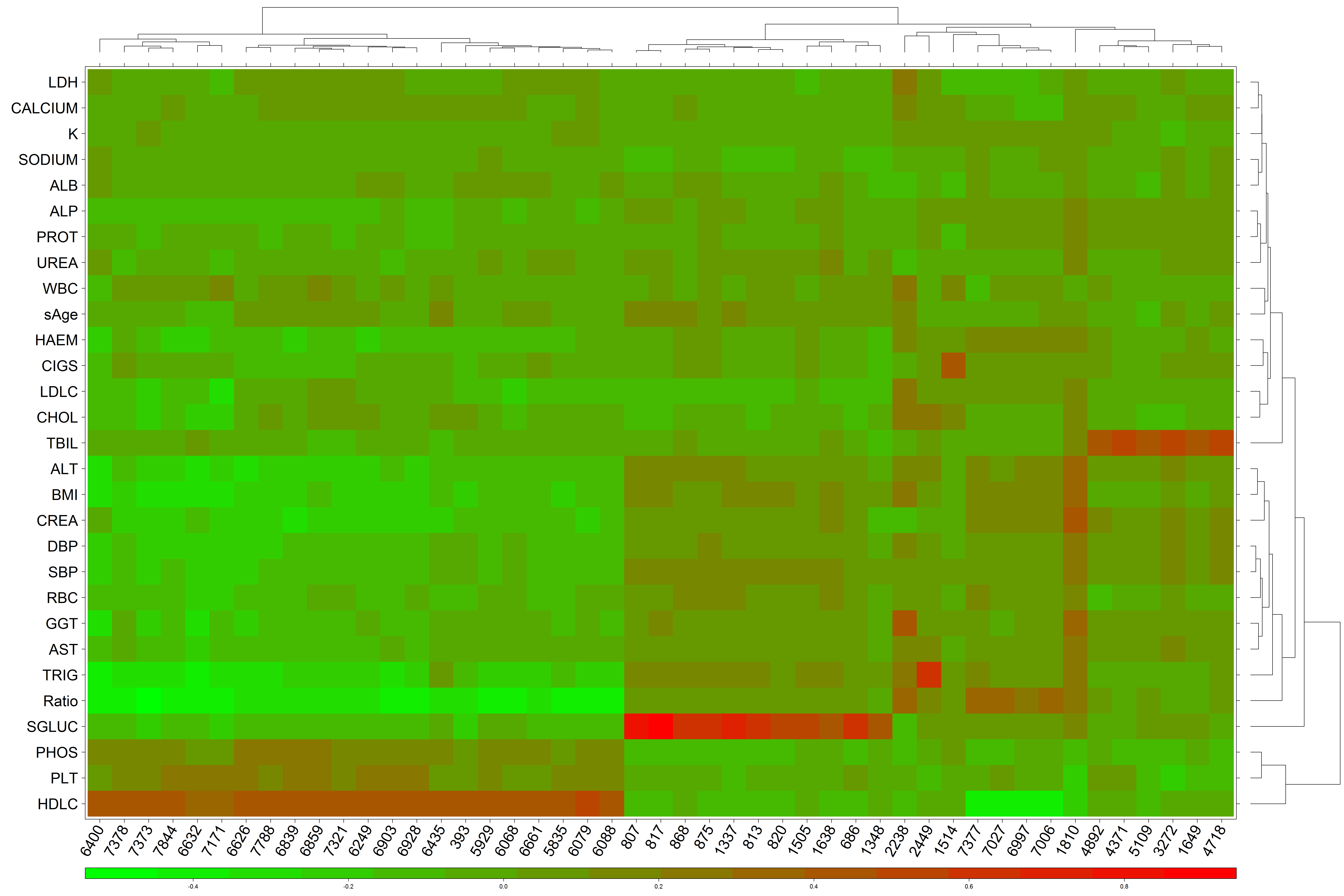


| Idx | Metabolite |
| --- | --- |
| 393 | Nitro-Cresol or Aminosalicylic acid or Hydroxylaminobenzoate |
| 686 | 3,3-Dimethylglutaric acid or 3-Methyladipic acid |
| 807 | Carbohydrate-based metabolite |
| 813 | Carbohydrate-based metabolite |
| 817 | Carbohydrate-based metabolite |
| 820 | Carbohydrate-based metabolite |
| 868 | Carbohydrate-based metabolite |
| 875 | Carbohydrate-based metabolite |
| 1337 | Allopurinol riboside |
| 1348 | 5-Methyldeoxycytidine |
| 1505 | 3''-Deamino-3''-oxonicotianamine or N-Glucosylnicotinate |
| 1514 | Glycerophosphocholine |
| 1638 | N6,N6-Dimethyladenosine or 1D-1-Guanidino-3-amino-1,3-dideoxy-scyllo-inositol 4-phosphate or 1D-1-Guanidino-3-amino-1,3-dideoxy-scyllo-inositol 6-phosphate |
| 1649 | 4-Hydroxy-3-polyprenylbenzoate or N-Caffeoylputrescine or L-isoleucyl-L-proline or L-leucyl-L-proline |
| 1810 | Uric acid |
| 2238 | L-Tyrosyl-L-arginine |
| 2449 | tetracosadienoic acid or MG(18:1) |
| 3272 | Leukotriene E3 or N-acetylsphingosine 1-phosphate |
| 4371 | 15,16-Dihydrobiliverdin OR Bilirubin |
| 4718 | 26,27-diethyl-1alpha,25-dihydroxy-22-thia-20-epivitamin D3 or 26,27-diethyl-1alpha,25-dihydroxy-22-thiavitamin D3 |
| 4892 | Mesobiliverdin Iv Alpha |
| 5109 | Urobilin |
| 5835 | Ubiquinone-8 or SM(d18:0/16:0) or CE(18:0) |
| 5929 | PC(O-14:0/16:0) |
| 6068 | Ubiquinol 8 or SM(d18:0/18:1) or CE(20:1) |
| 6079 | PE(20:2/dm18:1) or PE(20:3/dm18:0) |
| 6088 | SM(d18:0/18:0) or CE(20:0) |
| 6249 | PE(38:2) or PC(O-16:0/O-18:0) or GalCer(d18:1/18:1) or GluCer(d18:1/18:1) |
| 6400 | DG(46:7) or CE(22:5) |
| 6435 | PE(40:5) or PE(36:0) or PG(36:1) or GalCer(d18:1/20:0) or GluCer(d18:1/20:0) |
| 6626 | PE(40:2) or DG(46:3) |
| 6632 | PC(18:0/dm18:1) or PC(18:1/dm18:0) or PC(18:2/O-18:0) or PC(20:1/dm16:0) or PC(P-18:0/18:1) |
| 6661 | PE(42:5) or PE(40:2) or PE(38:0) or GalCer(d18:1/22:0) or GluCer(d18:1/22:0) |
| 6839 | PC(40:0) |
| 6859 | TG(52:7) or TG(50:4) or PE(42:0) or TG(48:1) or DG(46:0) or docosanyl octacosanoate or dotriacontanyl octadecanoate or octacosanyl docosanoate or tetratriacontanyl hexadecanoate or triacontanyl icosanoate |
| 6903 | SM(d18:0/24:0) or PC(40:4) |
| 6928 | docosanyl octacosanoate or dotriacontanyl octadecanoate or octacosanyl docosanoate or tetratriacontanyl hexadecanoate or triacontanyl icosanoate |
| 6997 | PC(38:2) or PC(36:1) |
| 7006 | PC(42:9) or PG(40:4) or DG(46:4) |
| 7027 | PC(42:8) or SM(d18:1/22:1) or DG(46:3) or TG(48:3) |
| 7171 | PC(44:3) or PC(42:0) or PC(24:0/dm18:0) |
| 7321 | PC(46:7) or PC(44:4) or PC(O-18:0/22:0) |
| 7373 | TG(58:12) or TG(56:9) |
| 7377 | PC(44:8) |
| 7378 | PC(24:0/dm18:1) or PC(24:1/dm18:0) |
| 7788 | PC(44:5) or PC(24:0/dm18:1) |
| 7844 | Galabiosylceramide (d18:1/22:0) or Lactosylceramide (d18:1/22:0) |

**Supplementary Figure 6** – Selection of feature subset by classification. Only the top 50 features are shown here for age ( age<50 and age>65), bmi(bmi<25 and bmi>30) and gender (male and female) in three analytical platforms GC-MS, UPLC-MS(+) and UPLC-MS(-). The re-sampling method, bootstrap, was used for evaluating the classification accuracy by random forest (RF).


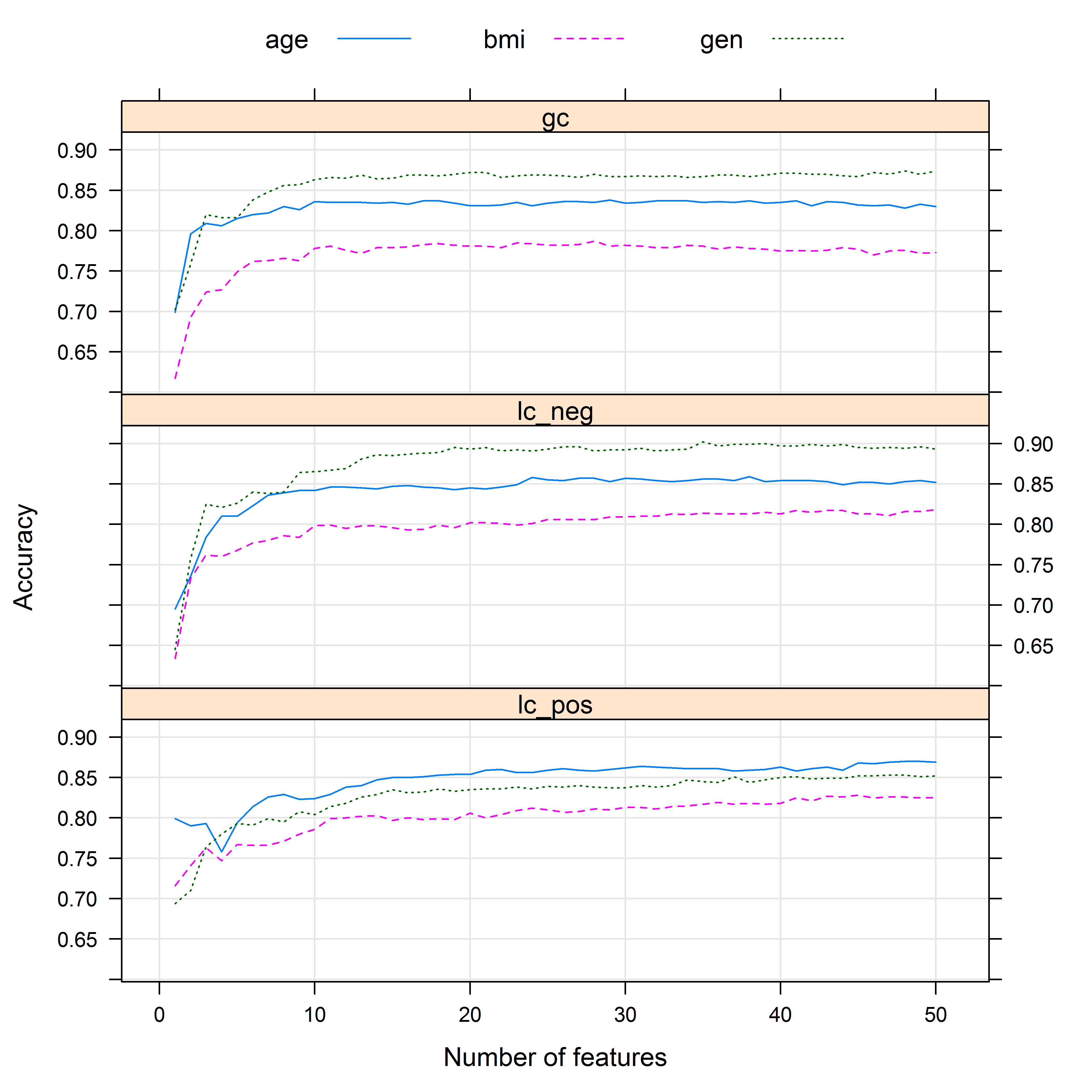


**Supplementary Figure 7** - Comparison of classification with and without feature selection. Two classifiers, Random forest (RF) and Support Vector Machine (SVM), were used for discrimination of three groups: age( age<50 and age>65), bmi (bmi<25 and bmi>30) and gender (male and female). The results were based on the average of 100 results by bootstraps re-sampling. Three analytical platforms, GC-MS, LC-MS(+) and LC-MS(-), were used.


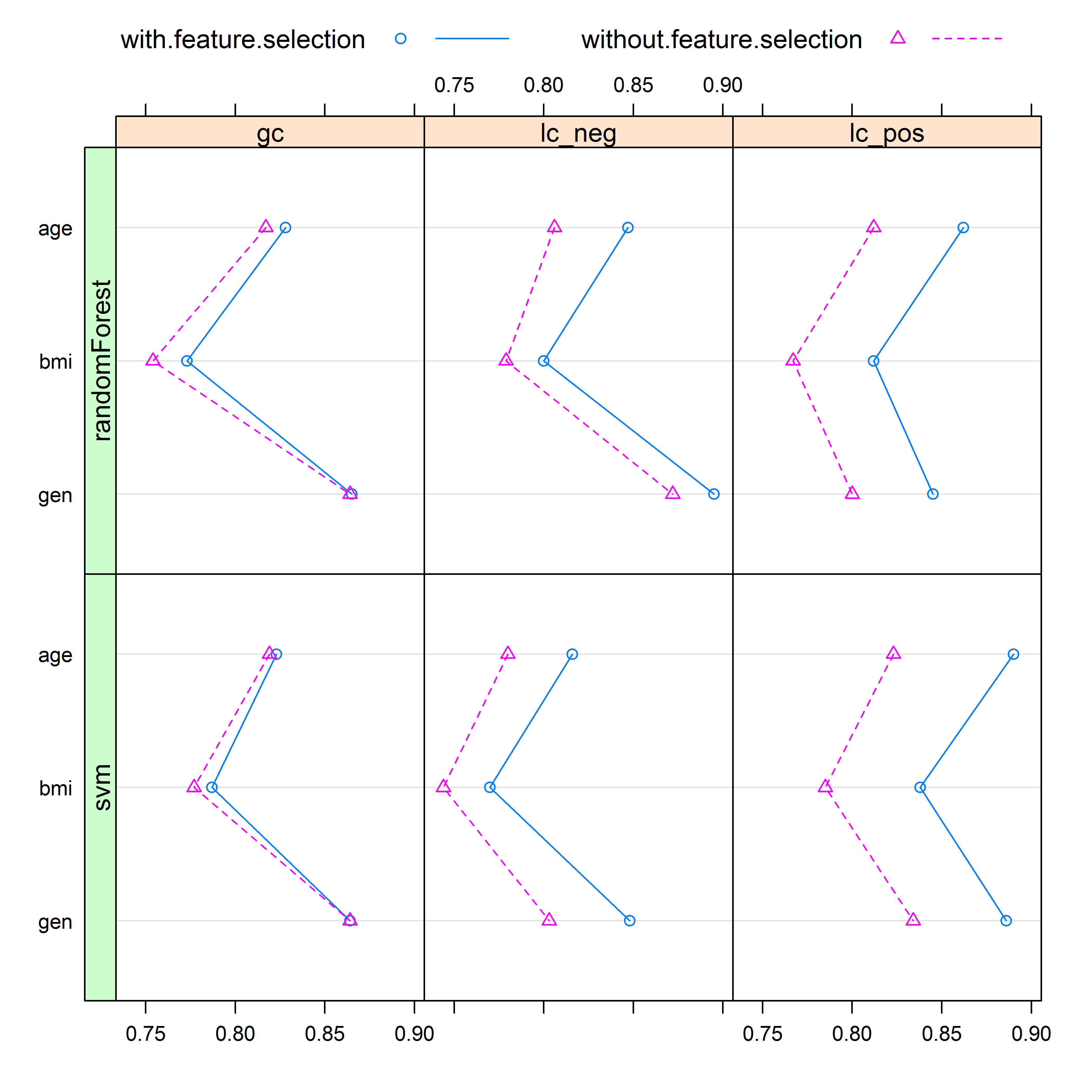

Supplement: Supplementary file 1 — Supplementary material 1 (DOC 8536 kb) [file 11306_2014_707_MOESM1_ESM.doc]
